# Mendelian randomization supports the causal role of fasting glucose on periodontitis

**DOI:** 10.3389/fendo.2022.860274

**Published:** 2022-08-05

**Authors:** Yi Wang, Tengda Chu, Yixuan Gong, Sisi Li, Lixia Wu, Lijian Jin, Rongdang Hu, Hui Deng

**Affiliations:** ^1^ Department of Orthodontics, School and Hospital of Stomatology, Wenzhou Medical University, Wenzhou, China; ^2^ Department of Periodontics, School and Hospital of Stomatology, Wenzhou Medical University, Wenzhou, China; ^3^ Division of Periodontology and Implant Dentistry, Faculty of Dentistry, The University of Hong Kong, Hong Kong, Hong Kong SAR, China

**Keywords:** Mendelian randomization, periodontitis, glycemic trait, T2D (type 2 diabetes), causal association

## Abstract

**Purpose:**

The effect of hyperglycemia on periodontitis is mainly based on observational studies, and inconsistent results were found whether periodontal treatment favors glycemic control. The two-way relationship between periodontitis and hyperglycemia needs to be further elucidated. This study aims to evaluate the causal association of periodontitis with glycemic traits using bi-directional Mendelian randomization (MR) approach.

**Methods:**

Summary statistics were sourced from large-scale genome-wide association study conducted for fasting glucose (N = 133,010), HbA1c (N = 123,665), type 2 diabetes (T2D, N = 659,316), and periodontitis (N = 506,594) among European ancestry. The causal relationship was estimated using the inverse-variance weighted (IVW) model and further validated through extensive complementary and sensitivity analyses.

**Results:**

Overall, IVW showed that a genetically higher level of fasting glucose was significantly associated with periodontitis (OR = 1.119; 95% CI = 1.045–1.197; *P*_FDR_= 0.007) after removing the outlying instruments. Such association was robust and consistent through other MR models. Limited evidence was found suggesting the association of HbA1C with periodontitis after excluding the outliers (IVW OR = 1.123; 95% CI = 1.026–1.229; *P*_FDR_= 0.048). These linkages remained statistically significant in multivariate MR analyses, after adjusting for body mass index. The reverse direction MR analyses did not exhibit the causal association of genetic liability to periodontitis with any of the glycemic trait tested.

**Conclusions:**

Our MR study reaffirms previous findings and extends evidence to substantiate the causal effect of hyperglycemia on periodontitis. Future studies with robust genetic instruments are needed to confirm the causal association of periodontitis with glycemic traits.

## Introduction

Periodontal disease, covering gingivitis and periodontitis, is a group of immuno-inflammatory disorders initiated by oral microbial dysbiosis, and if left untreated, periodontitis will lead to the destruction of tooth-supporting tissues and eventually tooth loss (1). As a highly prevalent non-communicable disease (NCD), severe periodontitis afflicts 10%–15% of the population worldwide, remaining a substantial socioeconomic burden (2–4).

Diabetes, characterized by poor glycemic control, is another common NCD with an estimated global prevalence of 9.3% (5). Prolonged hyperglycemia has been associated with a number of macrovascular and microvascular complications, leading to the damage of the corresponding organs such as heart, kidney, eyes, foot, and periodontal tissues (6, 7). Indeed, a two-way relationship has been proposed between diabetes and periodontitis (8). Diabetes is considered a risk for the development and progression of periodontitis. However, this evidence is mainly derived from observational studies, so that the causal inference is difficult to be established (9, 10). Moreover, the dose–response effect of fasting glucose and HbA1c on periodontitis has not been investigated. As for the reverse direction, the potential benefits of periodontal treatment on glycemic control remain unclear. Previous meta-analyses summarized that periodontal treatment reduced an average HbA1c level by 0.29% at 3–4 months, whereas such effect was not maintained after 4 months. Moreover, the conclusion should be interpreted with caution as the RCTs included are at high risk of bias due to the small sample size (11). Taken together, additional evidence for the causal inference on the two-way relationship between glycemic traits and periodontitis is highly warranted.

Two-sample Mendelian randomization (MR) is a powerful tool to explore the causal association in putative exposure–outcome pathways. It employs the genetic variants as unconfounded proxies for exposures to examine their effects on the outcome of interest (12). Because the genetic variants are randomly allocated at conception, MR is less vulnerable to confounding and reverse causation than observational studies. Furthermore, unlike RCT that describes the effect of an intervention in a certain period of time, MR assesses the impact of lifetime exposure to a genetic variant (13). In recent years, the increase in the accessible genome-wide association study (GWAS) data has fostered MR studies to employ single-nucleotide polymorphisms (SNPs) as instrumental variables (IVs) to estimate the causal effect of a modifiable risk factor on the disease, which supports or contradicts with previous epidemiological evidence (14). Recent MR studies have shown that genetically liability to periodontitis was not associated with glycemic control and type 2 diabetes (T2D) (15, 16). However, only study-level SNPs of periodontitis was adopted as proxy in this investigation, and the impact of glycemic traits such as glucose and HbA1c on periodontitis was not evaluated. In the present study, we investigated the relationship between periodontitis and glycemic trait (i.e., fasting glucose, HbA1c, and T2D) within the framework of bi-directional two-sample MR. The SNPs of both exposure and outcome were sourced from summary statistics of meta-analysis for GWAS in European ancestry populations.

## Materials and methods

### Study design

**Figure 1:**
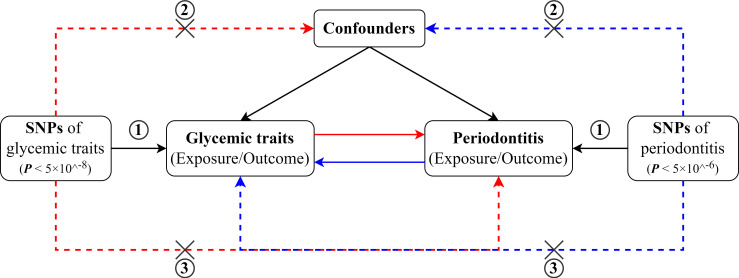
Schematic diagram for the bidirectional MR study on the associations between periodontitis and glycemic traits (fasting glucose, HbA1c, and T2D).

The overall study design for the bi-directional two-sample MR analyses has been illustrated in **Figure 1**. MR analyses depend on three core assumptions (17). The genetic variant for IV (1) has to be strongly correlated with the exposure of interest, known as the “relevance” assumption; (2) should not be associated with any confounders of the exposure–outcome linkage, known as the “independence assumption”; and (3) can only affect the outcome through the exposure, also known as the “exclusion-restriction” assumption, which means no pleiotropy. By using the summary-level statistics from GWAS, this two-sample MR study assessed the effects of glycemic traits on periodontitis and then the reverse direction (i.e., periodontitis on glycemic traits). This study is conducted following the STROBE-MR (Strengthening the Reporting of Observational studies in Epidemiology - Mendelian randomization) statement (18).

### Data sources

The characteristics of the data sources are described in **Supplemental Table 1**. All participants included in the original GWAS were of European descent. Summary statistics for periodontitis were derived from the largest meta-analyses consisting of sources from Gene-Lifestyle Interactions in Dental Endpoints (GLIDE) consortium and UK Biobank (UKB) (19). The SNPs were associated with the combined trait consists clinically defined periodontitis from GLIDE (N_cases_ = 17,353, N_controls_ = 28,210) and self-reported loose teeth from UKB (N_cases_ = 18,979, N_controls_ = 442,052). Periodontitis cases were classified either by the Centers for Disease Control and Prevention/American Academy of Periodontology definition, similar criteria evaluated by probing depth (20), or self-reported. Summary statistics of glycemic traits including fasting glucose and HbA1c were retrieved from Meta-analyses of Glucose and Insulin-Related Traits Consortium (MAGIC), which is the largest publicly available meta-analyses conducted in individuals without diabetes (https://magicinvestigators.org). We exclude the glycemic traits from patients with diabetes because anti-diabetic medication or insulin may affect glucose metabolism beyond genetic influence. The genetic data of fasting glucose and HbA1c have been reported with (21) and without body mass index (BMI) adjustment (22, 23). Because the use of covariable-adjusted summary associations could have bias the MR estimates, summary level data for fasting glucose (N = 133,010) and HbA1c (N = 123,665) without BMI adjustment were selected. The dataset for type 2 diabetes was retrieved from DIAbetes Genetics Replication and Meta-analyses (DIAGRAM) and Genetic Epidemiology Research on Aging (GERA) with N_Cases_ = 62,892 and N_Controls_ = 596,424 (24). The details can be found in their original publications.

### Selection of instrumental variables

To satisfy the first MR assumption, SNPs included have to be robustly associated with the exposure of interest at a genome-wide significant threshold (*P* < 5 × 10^−8^). Linkage disequilibrium (LD) clumping was performed to ensure the SNPs are independent (LD R^2^ < 0.001, LD distance > 10,000 kb). After selection, a total of 33 SNPs of fasting glucose, 37 of HbA1c, and 120 of T2D were obtained as IV candidates. Because only one genome-wide significant SNP was identified for the combined trait of periodontitis–loose teeth, an arbitrary threshold for suggestive association (*P* < 5 × 10^−6^) was adopted, and 22 SNPs were selected accordingly.

We next removed exposure-related SNPs that cannot be proxied in the outcome dataset. Palindromic SNPs were excluded due to the difficulty in identifying the effect alleles in the exposure and outcome. Those SNPs showing direct associations with both exposure and outcome (*P* < 5 × 10^−8^) were excluded to reduce the potential pleiotropy (25). F statistics of each SNP were calculated with the formula $\frac{R^{2}(N-2)}{\left(1-R^{2}\right)}$ to evaluate the strength of instruments (26, 27). SNPs with F < 10 were considered weak strength and removed.

### Statistical analyses

The method of MR was conducted using R (version 4.1.0), through “TwoSampleMR (version 0.5.6)”, “MendelianRandomization (version 0.5.1)”, and “MR-PRESSO (version 1.0)” R packages. The priori statistic power was calculated using the online platform (28) (https://sb452.shinyapps.io/power/). The genetic correlation and latent causal variable (LCV) were calculated using the Complex-Traits Genetics Virtual Lab (https://vl.genoma.io/).

To evaluate whether periodontitis and glycemic traits are genetically correlated, we performed cross-trait LD score regression with GWAS summary statistics using the Complex-Traits Genetics Virtual Lab (29). Prior to MR analyses, the summary statistics were harmonized so that the effect allele of each SNP was consistent between exposure and outcomes. Inverse-variance weighted (IVW) model was adopted as the primary analysis because it is a meta-approach combining Wald estimates for each SNP, effectively treating each SNP as a valid natural experiment. Importantly, it forces a zero intercept in the regression slope so that the result could be biased if any IV is invalid (30, 31). Three additional MR models were adopted, i.e., MR-Egger regression (32), weighted median, and weighted mode (33), which tolerate the existence of horizontal pleiotropy but have lower statistical power than IVW. LCV model is an MR-equivalent method to distinguish genetic correlation from causation, and it is robust to sample overlap (34). We used LCV to determine whether the causal association of glycemic traits with periodontitis could be explained by the genetic correlation. MR-Egger regression intercept was evaluated to estimate the presence of horizontal pleiotropy. Intercept centered at the origin with 95% CI including the null indicates the absence of pleiotropy. Furthermore, Cochran’s Q test was used to assess the heterogenicity among SNPs in IVW estimates (*P* < 0.05 indicates significant heterogenicity). IVW random effect model was performed if statistically significant heterogenicity was found. Otherwise fixed effect model was adopted (35). If MR-Pleiotropy RESidual Sum and Outlier (MR-PRESSO) (36) detected heterogenicity, then we would remove the outlying variants (*P* < 0.05 in the MR-PRESSO outlier test) and perform the MR analyses again. If heterogenicity was still statistically significant (indicated by Cochran’s Q test), then SNPs with *P* < 1 in the MR-PRESSO outlier test would be further excluded before the MR analyses (37). The influential SNPs were evaluated on the basis of several plots (leave-one-out, funnel plot, forest plot, and scatter plot). To correct for multiple comparisons, we applied false discovery rate (FDR) correction in the final *P*-value estimates. An FDR-corrected *P-*value (*P*_FDR_) <5% was considered significant, and an unadjusted *P*-value < 0.05 was considered the evidence of a suggestive association.

For SNPs used as instruments variables, we extracted R^2^ (variance in exposure traits explained by SNPs) if provided in the original datasets. Otherwise, the following formula was used to calculate R^2^ (27).


$$\frac{2\beta^{2}EAF\left(1-EAF\right)}{2\beta^{2}EAF\left(1-EAF\right)+{\left(se\left(\beta \right)\right)}^{2}2NEAF\left(1-EAF\right)}$$

To further verify the second assumption, we searched all the IVs in PhenoScanner (http://www.phenoscanner.medschl.cam.ac.uk) for association with previously reported confounders (*P* < 5 × 10^−8^) (38). We found IVs of BMI, which has been found to be associated hyperglycemia and periodontitis. We excluded these IVs of BMI in a sensitivity model and re-analyzed the MR estimates to gain a more direct cause-effect association. In addition, multivariate MR analysis was also conducted to minimize the correlated pleiotropy, which could have been introduced by indirect pathway (39). Summary statistics for BMI (N = ~700,000) were sourced from GIANT consortium (40).

The causal effects (i.e., T2D on periodontitis and vice versa) were converted from logit-scale to liability-scale using the following formula:


$$\beta_{XYliability}=\frac{Z_{KX}K_{Y}\left(1-K_{Y}\right)}{Z_{KY}K_{X}\left(1-K_{X}\right)}\beta_{XY}\log it$$

where *K_X_* and *Ky* are the population prevalence of exposure and outcome, and *Z_KX_* and *Z_KY_* are the height of standard normal distribution at such prevalence (41).

## Results

### Genetic correlation between glycemic traits and periodontitis

LD score regression showed statistically significant positive genetic correlations between periodontitis and fasting glucose (r_g_ = 0.226; *P* = 0.005), HbA1c (r_g_ = 0.198; *P* = 0.002), and T2D (r_g_ = 0.296; *P* = 4.47 × 10^−10^) (**Supplemental Table 2**). In addition, LCV estimates provided a genetic causality proportion (GCP) at −0.53 (*P* = 1 × 10^−6^), suggesting a possible (GCP < 0.6) genetic causation of HbA1c with periodontitis. On the other hand, LCV showed no evidence suggesting the genetic causation of periodontitis with fasting glucose (GCP = 0.02; *P* = 0.619) and T2D (GCP = 0.43; *P* = 0.283) (**Supplemental Table 3**).

### Causal effects of glycemic traits on periodontitis

The stringent selection process of IVs of glycemic traits on periodontitis has been described in **Supplemental Figure 1**. Following the screening, SNPs associated with fasting glucose (n = 30), HbA1c (n = 37), and T2D (n = 117) were included as valid IV. The minimum F statistics of these IVs were all larger than 10 (range from 28.654 to 29.942), ensuring the “relevance” assumption that the weak instrument bias was not likely to influence the estimations of causal effects. The characteristics of all the SNPs included as IVs of glycemic traits were detailed in **Supplemental Tables 4–6**. Our MR analyses are sufficiently powered to detect the association of fasting glucose (88%), HbA1c (75%), and T2D (93%) with an OR of 1.1 and a type 1 error rate of 0.05 (**Supplemental Table 7**).

**Figure 2:**
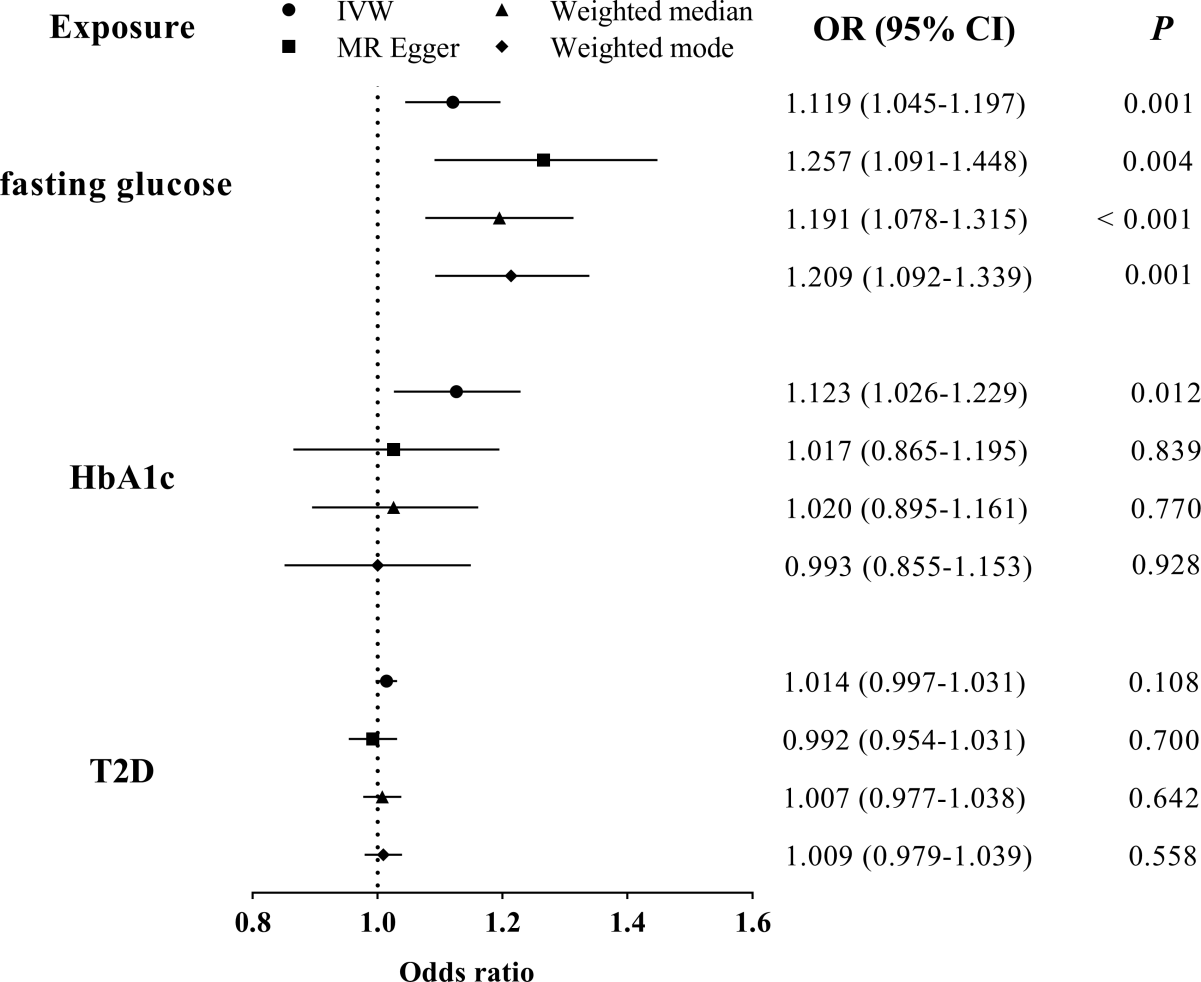
MR analyses for the causal association of glycemic traits (fasting glucose, HbA1c, and T2D) with periodontitis. IVW, inverse-variance weighted; T2D, type 2 diabetes. The error bar indicates the 95% confidence interval.

MR-PRESSO identified that rs6072275 and rs1280 were extreme outlying variants, which could markedly drive the MR association of fasting glucose with periodontitis. Following the removal of these two outliers, heterogeneity was no longer detected (Q = 27.872; *P* = 0.418). In addition, IVW showed a significant association [odds ratio (OR) = 1.119; 95% CI = 1.045–1.197; *P*_FDR_ = 0.007], suggesting that per 1-standard deviation (0.73 mmol/L) increment in fasting glucose was associated with higher OR of periodontitis. Moreover, such association was consistent and significant through all the other MR methods including MR-Egger (OR = 1.257; 95% CI = 1.091–1.448; *P*_FDR_ = 0.020), weight median (OR = 1.191; 95% CI = 1.078–1.315; *P*_FDR_ = 0.007), and weighted mode (OR = 1.209; 95% CI = 1.092–1.339; *P*_FDR_ = 0.007). Egger intercepts suggested no evidence of pleiotropy (*P* = 0.076). Taken together, these results suggested a causal association of genetically proxied higher fasting glucose with periodontitis. As for the association of HbA1c with periodontitis, we noted that rs11964178 and rs8192675 were extreme outlying IVs through MR-PRESSO. However, heterogenicity still existed (Q = 58.261; *P* = 0.008) after the removal of these two outliers. We further removed outliers with P-value less than 1 in the MR-PRESSO outlier test (rs560887, rs1387153, and rs17533903). Heterogenicity was no longer detected (Q = 29.907; *P* = 0.522), and pleiotropy was not found based on Egger intercept (*P* = 0.147). IVW results showed a significant association of HbA1c with periodontitis (OR = 1.123; 95% CI = 1.026–1.229; *P*_FDR_ = 0.048). Such association was not supported by MR-Egger, weighted median, and weighted mode, suggesting limited evidence supporting the causal association of HbA1c with periodontitis. In addition, our results suggest a null association of T2D on periodontitis through any of the MR models (**Table 1**, **Figure 2**).

**Table 1:** Summary statistics for MR analyses of the potential causal effect of glycemic traits on periodontitis.

Exposure	OR (95% CI)	*P*	*P* for FDR	*P* for Q-test	*P* for Intercept
**Fasting glucose**					
IVW	1.119 (1.045, 1.197)	0.001	0.007	0.418	0.076
MR-Egger	1.257 (1.091, 1.448)	0.004	0.020		
Weighted median	1.191 (1.078, 1.315)	6×10^-4^	0.007		
Weighted mode	1.209 (1.092, 1.339)	0.001	0.007		
**HbA1c**					
IVW	1.123 (1.026, 1.229)	0.012	0.048	0.522	0.147
MR-Egger	1.017 (0.865, 1.195)	0.839	0.883		
Weighted median	1.020 (0.895, 1.161)	0.770	0.856		
Weighted mode	0.993 (0.855, 1.153)	0.928	0.928		
**T2D**					
IVW	1.014 (0.997, 1.031)^*^	0.108	0.270	0.054	0.297
MR-Egger	0.992 (0.954, 1.031)^*^	0.700	0.824		
Weighted median	1.007 (0.977, 1.038)^*^	0.642	0.803		
Weighted mode	1.009 (0.979, 1.039)^*^	0.558	0.797		

FDR, false discovery rate; T2D, type 2 diseases; OR, odds ratio; IVW, inverse-variance weighted; Q-test: Cochran’s Q statistic; ^*^, liability scale.

**Figure 3:**
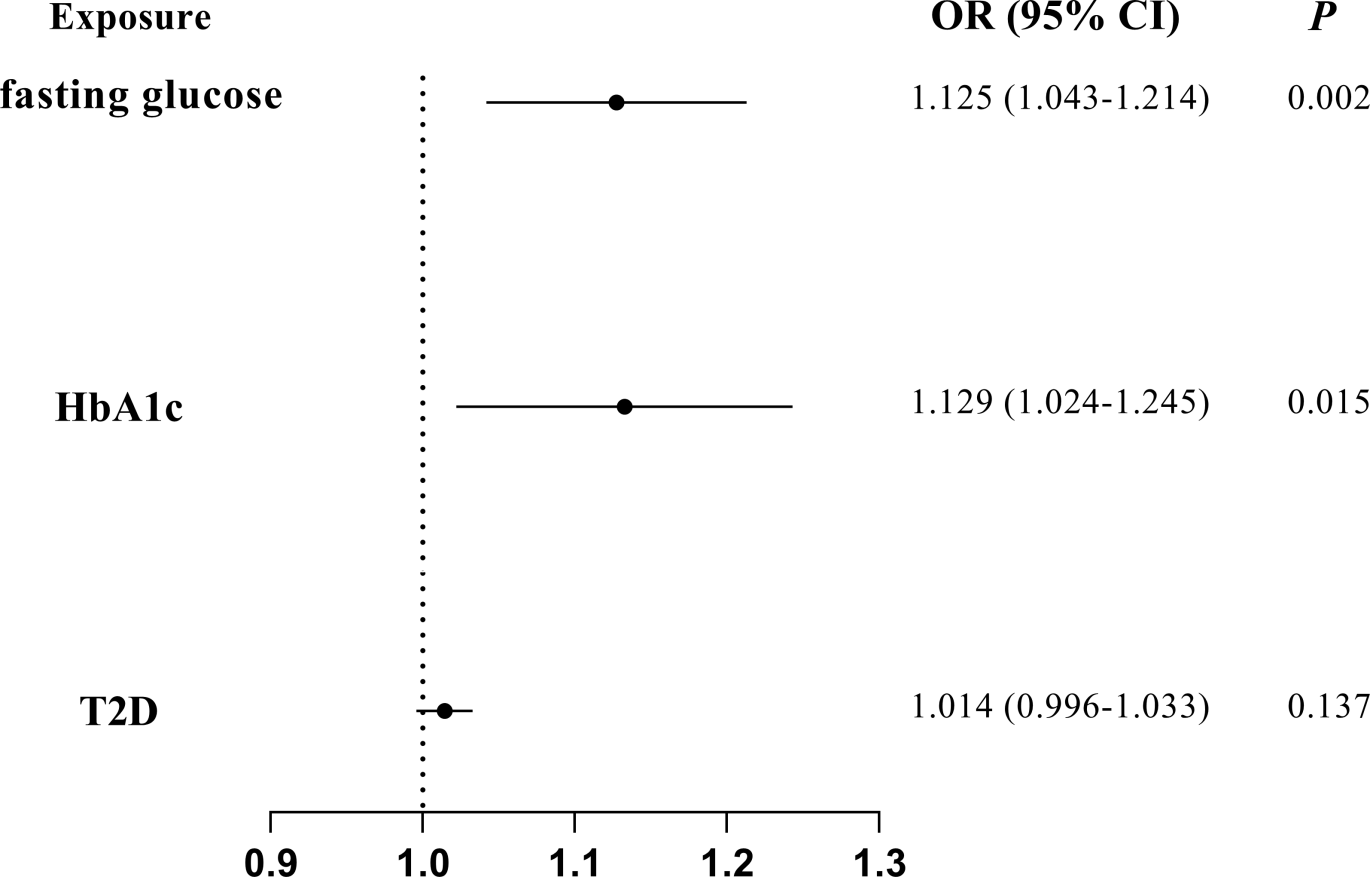
MR analyses on the causal association of glycemic traits (fasting glucose, HbA1c, and T2D) with periodontitis after excluding SNPs of BMI. T2D, type 2 diabetes. The error bar indicates the 95% confidence interval.

**Figure 4:**
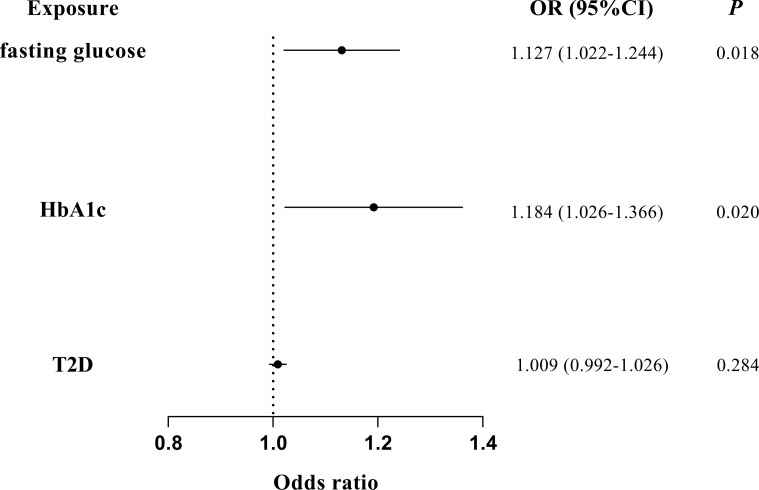
Multivariate MR analyses on the causal association of glycemic traits (fasting glucose, HbA1c, and T2D) with periodontitis by adjusting for BMI. T2D, type 2 diabetes. The error bar indicates the 95% confidence interval.

The associations of glycemic traits with periodontitis were further tested by removing IVs of BMI and multivariate MR to further reduce the potential horizontal pleiotropy introduced by confounders. The characteristics of the SNPs of BMI have been listed in **Supplemental Table 8**. We found a significant association of fasting glucose (IVW OR = 1.125; 95% CI = 1.043–1.214; *P* = 0.002) and HbA1c (IVW OR = 1.129; 95% CI = 1.024–1.245; *P* = 0.015) with periodontitis after excluding IVs of BMI (**Figure 3**). Furthermore, multivariate MR estimates also indicated a significant association of fasting glucose (IVW OR = 1.127; 95% CI = 1.022–1.244; *P* = 0.018) and HbA1c (IVW OR = 1.184; 95% CI = 1.026–1.366; *P* = 0.020) with periodontitis when BMI was accounted for (**Figure 4**).

There was no SNP substantially dominating the MR estimates as indicated by the “leave-one-out test”. Variant-specific causal estimates were visualized in funnel plot, scatter plot, and forest plot, indicating no considerable heterogenicity among the SNPs of fasting glucose, HbA1c, and type 2 diabetes (**Supplemental Figures 2–4**).

### Causal effects of periodontitis on glycemic traits

The workflow for IV selection of periodontitis is described in **Supplemental Figure 5**. After rigorous screening, eight SNPs associated with periodontitis were identified as valid IVs on HbA1c and 12 SNPs on T2D, respectively. The minimum F statistics for these IVs is 20.903 (all above 10), indicating that the weak instrument bias is neglectable. The SNPs associated with periodontitis were not available in the fasting glucose dataset, so that MR analyses were not performed. The characteristics of all the SNPs as IVs of periodontitis on glycemic traits were detailed in **Supplemental Tables 9, 10**. With a type 1 error rate of 0.05, our MR analyses are insufficiently powered when OR of periodontitis on HbA1c (31%) and T2D (53%) is set at 1.1 but adequately powered when OR is 1.2 (HbA1c, 80%; T2D, 97%) (**Supplemental Table 7**).

**Figure 5:**
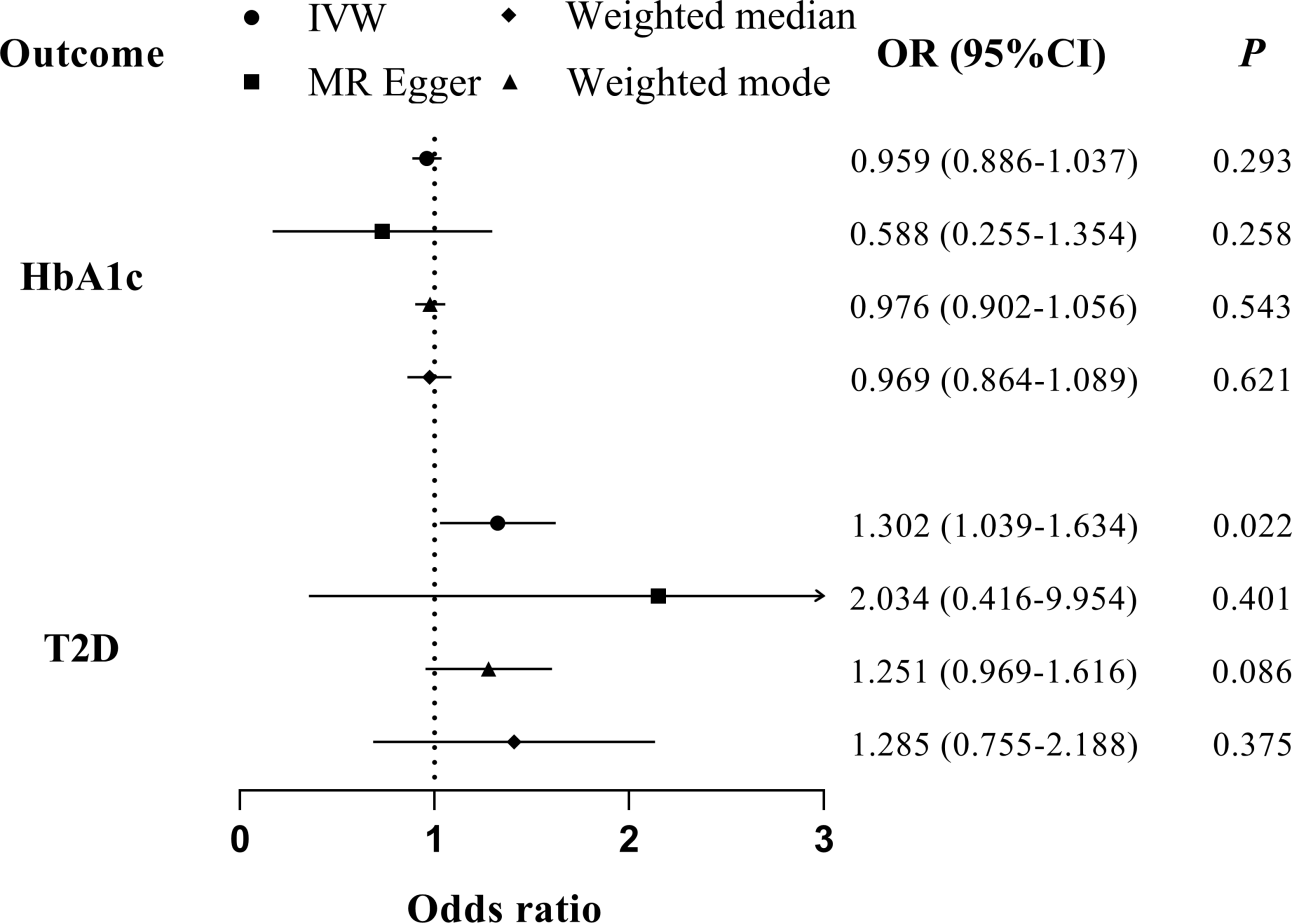
MR analyses for the causal association of periodontitis with glycemic traits (HbA1c and T2D). IVW, inverse-variance weighted; T2D, type 2 diabetes. The error bar indicates the 95% confidence interval.

We found no evidence suggesting the causal effects of periodontitis on glycemic traits through different MR methods (**Table 2**, **Figure 5**). Significant heterogenicity and pleiotropy were not detected, as indicated by Cochran’s Q statistics and Egger intercept. “leave-one-out test” analysis indicated no single SNP substantially driving the causal estimates. Variant-specific causal estimates were visualized in funnel plot, scatter plot, and forest plot, suggesting no considerable heterogenicity among the SNPs of periodontitis (**Supplemental Figures 6, 7**).

**Table 2:** Summary statistics for MR analyses of the potential causal effect of periodontitis on glycemic traits.

Outcome	OR (95% CI)	*P*	*P* for FDR	*P* for Q-test	*P* for Intercept
**HbA1c**					
IVW	0.959 (0.886, 1.037)	0.293	0.586	0.976	0.960
MR-Egger	0.588 (0.255, 1.354)	0.258	0.573		
Weighted median	0.976 (0.902, 1.056)	0.543	0.797		
Weighted mode	0.969 (0.864, 1.089)	0.621	0.803		
**T2D**					
IVW	1.302 (1.039, 1.634)^*^	0.022	0.073	0.052	0.590
MR-Egger	2.034 (0.416, 9.954)^*^	0.401	0.668		
Weighted median	1.251 (0.969, 1.616)^*^	0.086	0.246		
Weighted mode	1.285 (0.755, 2.188)^*^	0.375	0.668		

FDR, false discovery rate; T2D, type 2 diseases; OR, odds ratio; IVW, inverse-variance weighted; Q-test: Cochran’s Q statistic; ^*^, liability scale.

## Discussion

To the best of our knowledge, this is the first two-sample MR analysis investigating the bi-directional relationship between periodontitis and glycemic traits including fasting glucose, HbA1c, and T2D. By sourcing the summary-level data from previous GWAS, we found that genetically predisposition to a higher fasting glucose level is significantly associated with an increased risk of periodontitis. Limited evidence was found suggesting the association of HbA1c on periodontitis. Moreover, both fasting glucose and HbA1c retained a direct association with periodontitis in multivariate MR analyses adjusted for BMI. The reverse MR did not provide evidence supporting the causal inference of genetically proxied periodontitis on any of the glycemic traits of interest.

Epidemiological evidence from meta-analyses consisting of cross-sectional and longitudinal studies showed that hyperglycemia and T2D account for increased prevalence and severity of periodontitis, tooth loss, and edentulism (9). Although many of the above clinical studies have made adjustments for observed confounders, residual confounding may still exist to distort the association estimates. Moreover, causality is difficult to be obtained from these observational findings, and it is unethical and infeasible to conduct RCTs to determine the effects of hyperglycemia and diabetes on periodontitis. Unlike findings from the observational studies, the use of genetic variants as IVs in MR analyses is less vulnerable to modifiable confounders and reverse causality according to Mendel’s law of inheritance (13). Notably, our MR results extend previous literature by showing the pronounced association of fasting glucose on periodontitis. IVW estimates showed limited evidence supporting the association of HbA1c on periodontitis, likely due to the lower statistical power because our genetic instruments of HbA1c only explained 2.3% of the phenotypic variance, which is smaller than that of fasting glucose (3.2%). Another explanation is that the genetic loci affecting HbA1c through both glucose and erythrocytic pathways (23). The IVs associated with erythrocytic pathways might the limit the detection power. Considering that obesity is a common risk factor for hyperglycemia and periodontitis (42), we performed multivariate MR by adjusting BMI, further strengthening the directional causal association of fasting glucose and HbA1c on periodontitis. On the contrary to previous clinical findings, our results did not support the association of predisposition to T2D on the risk of periodontitis, which was in consistent with a recent MR study (16). The null association of T2D with periodontitis could be explained by the heterogenicity of T2D associated genetic loci (43). We hypothesized that the causal effect of T2D on periodontitis might arise from hyperglycemia, rather than insulin resistance or beta cell function.

Although the statistical significant causal association of fasting glucose with periodontitis was consistent across different MR methods, cautious interpretation on the clinical meaning of the MR estimates should be taken. It has to be noted that, like all other MR investigations, the causal estimates found in the current study should not be generally interpreted as the impact of intervening the glycemic traits on the risk of periodontitis in clinical practice. Instead, it should be better interpreted as a test statistic for a causal hypothesis, providing an alternative line of etiological evidence supporting the causal association of hyperglycemia with periodontitis (44, 45).

The association of periodontitis with systemic disease (e.g., cardiovascular disease, diabetes, and adverse pregnancy outcomes) has attracted considerable attention over the past decades. Although there has been a great progress in the mechanism underlying the periodontitis-systemic disease linkage, findings from RCTs testing the systemic effect of periodontal treatment are sometimes inconsistent and conflicting (46). A pooled estimate of about 0.3% reduction of HbA1c at 3 months following periodontal treatment has been noted in a Cochrane meta-analyses (11). However, inconsistent results were obtained from two RCTs with relatively large sample size in extended period. One (n = 264) showed a significant reduction of HbA1c at 12-month (−0.6%, 95% CI: −0.9% to −0.3%) and 6-month (−0.3%, 95% CI: −0.5% to 0.0%) follow-ups (47), whereas the other (n = 514) failed to detect a beneficial effect at 6-month (−0.05%, 95% CI: −0.23 to 0.12%) follow-ups (48). Moreover, a null effect was also observed in our earlier clinical trial (49). In the current study, the MR estimates did not show a robust causal association of periodontitis with any of the glycemic traits of interest, which is consistent with a previous MR using study-level SNPs (15). It has to be noted that null association could be explained by the lack of association strength of genetic variants for periodontitis. Although the estimated heritability of periodontitis is relatively high, GWAS of periodontitis defined by clinical criteria has had modest success to date, failing to identify consistent SNPs (50). In this meta-analysis GWAS dataset that we sourced, only one SNP reached genome-wide significance for the combined trait of periodontitis and loose teeth, whereas none for the single trait of periodontitis (19). A suggestive association (*P* < 5 × 10^−6^) was therefore adopted, but the genetic instruments only explained approximately 0.06% of the phenotypic variance. As such, we were not capable of detecting small effects although significant horizontal pleiotropy and weak instrument bias (all IVs with F statistics > 10) have been ruled out. In line with our findings, a recent MR study adopting genetic variants (n = 5) on the basis of single GWAS also found no causal effect of periodontitis on T2D. These genetic variants had a mean F of 4.89 (ranging from 0.895 to 7.294), suggesting a weak instrumental bias (16). Nevertheless, it is premature to conclude the null association of periodontitis with glycemic traits based on the current findings. Identifying more genetic instruments with robust association is highly warranted to boost the detection power in further MR studies using periodontitis as exposure.

A key strength of our study is that we sourced data of exposure and outcome from summary-level GWAS on different cohorts with large sample size. The effect size could be more precisely assessed with multiple sensitivity analyses in comparison to MR analyses using study-level data from a smaller cohort. Moreover, multiple sensitivity analyses were conducted to evaluate and minimize the potential heterogenicity and pleiotropy. In addition, horizontal pleiotropy was further reduced by the multivariate MR adjusting for BMI. Our study has several limitations. First, like all the other MR studies, the genetic variants represent lifelong exposure. The significant association found in our study is likely to reflect the life-long effect of hyperglycemia on periodontitis. Second, there are several overlapping cohorts contributing to the summary statistics of both exposure and outcome. However, the F statistics indicated that the included instruments are relatively strong, which could reduce the potential bias due to overlapping sampling (51). Third, although the analyses are restricted to European descents to minimize the bias from population stratification, our findings may not be generalized to other ethnicities. Fourth, the trait BMI is heterogeneous, which could bias the multivariate MR estimates. Because an increased BMI results from either excessive fat or fat-free mass, SNPs of BMI could be associated unfavorable adiposity or favorable adiposity, which may confer different metabolic effects on periodontitis (52).

In conclusion, we extended previous observational findings by demonstrating a significant causal association of genetically determined higher levels of fasting glucose with periodontitis, highlighting the importance of glycemic control for better oral/periodontal care. In the reverse direction, current results did not support a robust causal association of periodontitis with the glycemic traits of interest. Future GWAS identifying more genetic loci that strongly associated with periodontitis is needed to support or contradict this null association.

## Data availability statement

The original contributions presented in the study are included in the article/**Supplementary Material**. Further inquiries can be directed to the corresponding author/s.

## Ethics statement

This study only used publicly available data and the relevant ethical approval can be found in the corresponding studies referenced in the Materials and Methods section.

## Author contributions

YW, RH, and HD contributed to the conception and design of the study. YW and TC organized the database and performed the statistical analysis. YG, SL, and LW performed the statistical analysis. YW, TC wrote the first draft of the manuscript. LJ, RH, and HD refined the manuscript. All authors contributed to manuscript revision, read, and approved the submitted version.

## Funding

This study was financially supported by grants from the National Natural Foundation of China (No.82101029), Natural Science Foundation of Zhejiang Province (No. LQ20H140002), and Wenzhou Science and Technology Bureau (No. Y20190100 and ZY2020021). Zhejiang Provincial Public Welfare Science and Technology Project, Grant/Award Number: LGF21H140006.

## Acknowledgments

The authors are enormously grateful to the investigators of the original GWAS for sharing their summary level data used in this study.

## Conflict of interest

The authors declare that the research was conducted in the absence of any commercial or financial relationships that could be construed as a potential conflict of interest.

## Publisher’s note

All claims expressed in this article are solely those of the authors and do not necessarily represent those of their affiliated organizations, or those of the publisher, the editors and the reviewers. Any product that may be evaluated in this article, or claim that may be made by its manufacturer, is not guaranteed or endorsed by the publisher.
