# Supplementary material for: Mendelian randomization supports the causal role of fasting glucose on periodontitis
Source: Front Endocrinol (Lausanne). 2022 Aug 5;13:860274. doi: 10.3389/fendo.2022.860274 (PMC9388749; doi:10.3389/fendo.2022.860274)
Supplement: Supplementary file 1 [file DataSheet_1.docx]

**Supplementary materials**

***A Mendelian Randomization study on the bi-directional link between periodontitis and glycemic traits***

Yi Wang, Tengda Chu, Yixuan Gong, Sisi Li, Lixia Wu, Lijian Jin, Rongdang Hu, Hui Deng

**Supplemental Table 1** Characteristics of studies used for Primary MR analysis.

**Supplemental Table 2** Results of LD score regression for the genetic correlation between periodontitis and glycemic traits.

**Supplemental Table 3** Results of latent causal variable for the genetic causal effect between periodontitis and glycemic traits.

**Supplemental Table 4** Characteristics of the SNPs used as IVs in the causal association of fasting glucose with periodontitis.

**Supplemental Table 5** Characteristics of the SNPs used as IVs in the causal association of HbA1c with periodontitis.

**Supplemental Table 6** Characteristics of the SNPs used as IVs in the causal association of T2D with periodontitis.

**Supplemental Table 7** Priori power calculation for the association estimates in MR analyses.

**Supplemental Table 8** Characteristics of the SNPs of BMI.

**Supplemental Table 9** Characteristics of the SNPs used as IVs in the causal association of periodontitis with HbA1c.

**Supplemental Table 10** Characteristics of the SNPs used as IVs in the causal association of periodontitis with T2D.

**Supplemental Fig. 1** Flow chart describing the screening of valid instrumental variable selection of glycemic traits on periodontitis.

**Supplemental Fig. 2** Sensitivity analysis of MR estimates on the association of fasting glucose with periodontitis.

**Supplemental Fig. 3** Sensitivity analysis of HbA1c on periodontitis.

**Supplemental Fig. 4** Sensitivity analysis of T2D on periodontitis.

**Supplemental Fig. 5** Flow chart describing the screening of valid instrumental variable selection of periodontitis on glycemic traits

**Supplemental Fig. 6** Sensitivity analysis of periodontitis on HbA1c.

**Supplemental Fig. 7** Sensitivity analysis of periodontitis on T2D.

**Supplemental tables**

**Supplemental Table 1.** Characteristics of studies used for Primary MR analysis.

| Traits | Reference | Consortium | Sample size | Population |
| --- | --- | --- | --- | --- |
| Fasting glucose | Scott et al. | MAGIC | 133,010 | European |
| HbA1c | Wheeler E et al. | MAGIC | 123,665 | European |
| T2D | Xue et al. | PCTG | 659,316 | European |
| Periodontitis | Shungin et al. | GLIDE & UKB | 506,594 | European |

、

Abbreviations: HbA1c, hemoglobin A1c; T2D, type 2 diabetes; MAGIC, Meta-analyses of Glucose and Insulin-Related Traits Consortium; DIAGRAM, Diabetes Genetics Replication and Meta-analyses; UKB, UK biobank; PCTG, Program in complex trait Genomics.

**Supplemental Table 2.** Results of LD score regression for the genetic correlation between periodontitis and glycemic traits.

| **Trait 1** | **Trait 2** | **r_g_ (SE)** | ***P*** |
| --- | --- | --- | --- |
| Periodontitis | Fasting glucose | 0.226 (0.081) | 0.005 |
|  | HbA1c | 0.198 (0.065) | 0.002 |
|  | T2D | 0.296 (0.047) | 4.47×10^-10^ |

Abbreviations:HbA1c, hemoglobinA1c; T2D, type 2 diabetes; r_g_; the genetic correlation between the two traits,estimated by LD score regression.

**Supplemental Table 3.** Results of latent causal variable for the genetic causal effect between periodontitis and glycemic traits.

| **Trait 1** | **Trait 2** | **GCP (SE)** | ***P*** |
| --- | --- | --- | --- |
| Periodontitis | Fasting glucose | 0.02 (0.57) | 0.619 |
|  | HbA1c | -0.53 (0.17) | 1×10^-6^ |
|  | T2D | 0.43 (0.37) | 0.283 |

Abbreviations:HbA1c, hemoglobinA1c; T2D, type 2 diabetes; GCP; genetic causality proportion.

**Supplemental Table 4.** Characteristics of the SNPs used as IVs in the causal association of fasting glucose with periodontitis

| **SNP** | **EA** | **OA** | **EAF** | **beta** | **SE** | ***P*** | **R^2^** | **F statistics** |
| --- | --- | --- | --- | --- | --- | --- | --- | --- |
| rs10811661 | T | C | 0.829 | -0.024 | 0.003 | 5.65×10^-18^ | 0.00055 | 73.469 |
| rs10814916 | C | A | 0.519 | 0.016 | 0.002 | 2.26×10^-13^ | 0.00040 | 52.893 |
| rs11195502 | C | T | 0.927 | -0.032 | 0.004 | 1.97×10^-18^ | 0.00056 | 74.799 |
| rs11558471 | A | G | 0.691 | -0.029 | 0.002 | 7.80×10^-37^ | 0.00119 | 158.979 |
| rs11603334 | G | A | 0.856 | -0.019 | 0.003 | 1.12×10^-11^ | 0.00035 | 46.046 |
| rs11607883 | G | A | 0.470 | -0.021 | 0.002 | 6.32×10^-24^ | 0.00075 | 100.000 |
| rs11619319 | A | G | 0.777 | 0.020 | 0.002 | 1.33×10^-15^ | 0.00052 | 69.444 |
| rs11708067 | A | G | 0.784 | -0.023 | 0.003 | 1.30×10^-18^ | 0.00059 | 78.254 |
| rs11715915 | C | T | 0.686 | -0.012 | 0.002 | 4.90×10^-08^ | 0.00022 | 29.752 |
| rs1260326 | C | T | 0.575 | 0.029 | 0.0021 | 2.17×10^-41^ | 0.00143 | 190.703 |
| rs1280 | T | C | 0.867 | -0.026 | 0.003 | 5.04×10-^10^ | 0.00031 | 40.960 |
| rs12888855 | C | A | 0.782 | -0.016 | 0.003 | 5.04×10^-10^ | 0.00031 | 40.960 |
| rs16913693 | T | G | 0.977 | -0.043 | 0.007 | 3.51×10^-11^ | 0.00032 | 42.447 |
| rs17168486 | C | T | 0.832 | 0.031 | 0.003 | 3.17×10^-28^ | 0.00009 | 122.576 |
| rs174576 | C | A | 0.666 | -0.020 | 0.002 | 1.18×10^-18^ | 0.00062 | 82.645 |
| rs2191349 | G | T | 0.457 | 0.029 | 0.002 | 1.28×10^-42^ | 0.00143 | 190.702 |
| rs3829109 | G | A | 0.734 | -0.017 | 0.003 | 1.13×10^-10^ | 0.00030 | 39.643 |
| rs4502156 | T | C | 0.578 | -0.022 | 0.002 | 1.38×10^-25^ | 0.00082 | 109.751 |
| rs4869272 | C | T | 0.309 | 0.018 | 0.002 | 1.02×10^-15^ | 0.00050 | 66.942 |
| rs560887 | C | T | 0.709 | 0.071 | 0.003 | 1.40×10^-178^ | 0.00602 | 806.560 |
| rs6072275 | G | A | 0.846 | 0.016 | 0.003 | 1.66×10^-08^ | 0.00025 | 32.653 |
| rs6113722 | G | A | 0.955 | -0.035 | 0.005 | 2.49×10^-11^ | 0.00032 | 43.610 |
| rs6943153 | C | T | 0.703 | -0.015 | 0.002 | 1.63×10^-12^ | 0.00034 | 46.488 |
| rs6975024 | C | T | 0.173 | 0.061 | 0.003 | 2.88×10^-99^ | 0.00332 | 442.449 |
| rs749067 | C | T | 0.394 | -0.017 | 0.002 | 6.12×10^-15^ | 0.00045 | 59.711 |
| rs7651090 | A | G | 0.685 | 0.013 | 0.002 | 1.75×10^-08^ | 0.00024 | 31.947 |
| rs7903146 | C | T | 0.704 | 0.022 | 0.002 | 2.71×10^-20^ | 0.00063 | 84.028 |
| rs882020 | C | T | 0.854 | 0.021 | 0.003 | 3.04×10^-12^ | 0.00037 | 49.000 |
| rs9368222 | C | A | 0.736 | 0.014 | 0.002 | 1.00×10^-10^ | 0.00028 | 37.051 |
| rs983309 | G | T | 0.894 | -0.026 | 0.003 | 6.29×10^-15^ | 0.00047 | 62.075 |

Abbreviations: SNP, single nucleotide polymorphisms; EA, effect allele; OA, other allele; EAF, effect allele frequency; Beta, effect size; SE, standard error; R2, proportion of variance in exposure variable explained by SNP; F statistic, indicates the strength of the instrument variables. NA, not applicable.

**Supplemental Table 5.** Characteristics of the SNPs used as IVs in the causal association of HbA1c with periodontitis

| **SNP** | **EA** | **OA** | **EAF** | **beta** | **SE** | ***P*** | **R^2^** | **F statistics** |
| --- | --- | --- | --- | --- | --- | --- | --- | --- |
| rs1046896 | C | T | 0.686 | -0.028 | 0.002 | 4.46×10^-64^ | 0.00219 | 271.276 |
| rs10774625 | A | G | 0.456 | -0.009 | 0.002 | 1.46×10^-08^ | 0.00024 | 30.250 |
| rs11248914 | C | T | 0.314 | -0.014 | 0.002 | 2.56×10^-14^ | 0.00044 | 54.293 |
| rs11603334 | A | G | 0.129 | -0.012 | 0.002 | 6.85×10^-09^ | 0.00026 | 32.653 |
| rs11708067 | A | G | 0.774 | 0.013 | 0.002 | 1.42×10^-12^ | 0.00038 | 46.814 |
| rs11964178 | A | G | 0.585 | 0.010 | 0.002 | 6.38×10^-10^ | 0.00029 | 35.999 |
| rs12368284 | A | G | 0.612 | 0.012 | 0.002 | 2.92×10^-10^ | 0.00029 | 35.999 |
| rs12621844 | C | T | 0.373 | -0.010 | 0.002 | 1.87×10^-08^ | 0.00024 | 30.250 |
| rs13134327 | A | G | 0.310 | 0.013 | 0.002 | 2.64×10^-15^ | 0.00047 | 58.477 |
| rs13266634 | C | T | 0.761 | 0.015 | 0.002 | 4.53×10^-20^ | 0.00063 | 77.853 |
| rs1387153 | C | T | 0.728 | -0.019 | 0.002 | 2.11×10^-24^ | 0.00081 | 99.998 |
| rs1547247 | A | G | 0.252 | -0.014 | 0.002 | 1.73×10^-17^ | 0.00055 | 67.819 |
| rs17509001 | C | T | 0.155 | 0.018 | 0.002 | 1.94×10^-15^ | 0.00050 | 61.247 |
| rs17533903 | A | G | 0.265 | 0.015 | 0.002 | 5.27×10^-12^ | 0.00038 | 46.487 |
| rs17747324 | C | T | 0.192 | 0.015 | 0.002 | 6.12×10^-11^ | 0.00034 | 42.532 |
| rs1800562 | A | G | 0.053 | -0.040 | 0.004 | 4.67×10^-28^ | 0.00100 | 123.455 |
| rs2246434 | A | G | 0.310 | 0.019 | 0.002 | 1.99×10^-27^ | 0.00090 | 111.418 |
| rs2383208 | A | G | 0.792 | 0.014 | 0.002 | 7.04×10^-12^ | 0.00036 | 44.444 |
| rs267738 | G | T | 0.214 | -0.011 | 0.002 | 2.59×10^-09^ | 0.00027 | 33.517 |
| rs2979422 | C | T | 0.133 | 0.015 | 0.002 | 1.1×10^-10^ | 0.00034 | 42.532 |
| rs3782123 | A | C | 0.677 | -0.013 | 0.002 | 1.51×10^-10^ | 0.00034 | 42.249 |
| rs423117 | C | T | 0.879 | -0.019 | 0.003 | 1.3×10-^12^ | 0.00040 | 49.519 |
| rs4607517 | A | G | 0.195 | 0.031 | 0.002 | 8.76×10^-38^ | 0.00135 | 166.838 |
| rs4737009 | A | G | 0.270 | 0.021 | 0.002 | 4.48×10^-27^ | 0.00089 | 110.248 |
| rs4745982 | G | T | 0.085 | -0.095 | 0.006 | 2.87×10^-65^ | 0.00232 | 287.782 |
| rs560887 | C | T | 0.674 | 0.028 | 0.002 | 1.48×10^-58^ | 0.00195 | 241.971 |
| rs579459 | C | T | 0.204 | 0.011 | 0.002 | 9.42×10^-09^ | 0.00027 | 33.517 |
| rs592423 | A | C | 0.451 | 0.009 | 0.002 | 3.96×10^-08^ | 0.00023 | 28.654 |
| rs6474359 | C | T | 0.032 | -0.044 | 0.005 | 1.5×10^-16^ | 0.00056 | 68.920 |
| rs7040409 | C | G | 0.955 | 0.028 | 0.004 | 2.56×10^-14^ | 0.00046 | 57.267 |
| rs7616006 | A | G | 0.562 | 0.010 | 0.002 | 5.07×10^-10^ | 0.00028 | 34.602 |
| rs8192675 | C | T | 0.296 | -0.011 | 0.002 | 1.38×10^-11^ | 0.00034 | 41.868 |
| rs837763 | C | T | 0.482 | -0.017 | 0.002 | 1.68×10^-28^ | 0.00091 | 112.889 |
| rs855791 | A | G | 0.412 | 0.017 | 0.002 | 3.44×10^-28^ | 0.00091 | 112.889 |
| rs9818758 | A | G | 0.168 | 0.012 | 0.002 | 7.74×10^-10^ | 0.00029 | 35.999 |
| rs9914988 | A | G | 0.805 | 0.013 | 0.002 | 2.77×10^-11^ | 0.00034 | 42.249 |
| rs9935401 | A | G | 0.450 | 0.010 | 0.002 | 1.87×10^-08^ | 0.00024 | 30.250 |

Abbreviations: SNP, single nucleotide polymorphisms; EA, effect allele; OA, other allele; EAF, effect allele frequency; Beta, effect size; SE, standard error; R2, proportion of variance in exposure variable explained by SNP; F statistic, indicates the strength of the instrument variables. NA, not applicable.

**Supplemental Table 6.** Characteristics of the SNPs used as IVs in the association of T2D with periodontitis

| **SNP** | **EA** | **OA** | **EAF** | **beta** | **SE** | ***P*** | **R^2^** | **F statistics** |
| --- | --- | --- | --- | --- | --- | --- | --- | --- |
| rs10077431 | A | C | 0.215 | -0.049 | 0.009 | 4.45×10^-08^ | 0.00005 | 29.942 |
| rs10087241 | G | A | 0.405 | 0.048 | 0.008 | 2.89×10^-09^ | 0.00005 | 35.254 |
| rs10100265 | A | C | 0.390 | 0.049 | 0.008 | 5.13×10^-10^ | 0.00006 | 38.628 |
| rs10114341 | C | T | 0.441 | -0.041 | 0.007 | 1.34×10^-08^ | 0.00005 | 32.269 |
| rs10401969 | C | T | 0.077 | 0.092 | 0.013 | 4.37×10^-12^ | 0.00007 | 47.953 |
| rs1050226 | G | A | 0.407 | -0.049 | 0.007 | 3.24×10^-11^ | 0.00007 | 44.025 |
| rs1061813 | G | A | 0.463 | 0.043 | 0.007 | 4.18×10^-09^ | 0.00005 | 34.536 |
| rs1063355 | T | G | 0.398 | -0.071 | 0.008 | 2.84×10^-19^ | 0.00012 | 80.545 |
| rs10740322 | G | A | 0.313 | -0.048 | 0.009 | 2.00×10^-08^ | 0.00005 | 31.492 |
| rs10811661 | C | T | 0.174 | -0.157 | 0.010 | 1.08×10^-57^ | 0.00039 | 256.326 |
| rs10830963 | G | C | 0.276 | 0.091 | 0.008 | 6.43×10^-30^ | 0.00020 | 129.106 |
| rs10842994 | T | C | 0.197 | -0.076 | 0.009 | 1.07×10^-16^ | 0.00010 | 68.835 |
| rs10974438 | C | A | 0.351 | 0.059 | 0.008 | 3.27×10^-15^ | 0.00009 | 62.094 |
| rs11098676 | T | C | 0.212 | -0.054 | 0.010 | 1.86×10^-08^ | 0.00005 | 31.641 |
| rs11107116 | T | G | 0.220 | 0.047 | 0.009 | 3.93×10^-08^ | 0.00005 | 30.185 |
| rs11257655 | T | C | 0.207 | 0.074 | 0.009 | 2.43×10^-17^ | 0.00011 | 71.762 |
| rs1127655 | C | T | 0.471 | 0.044 | 0.008 | 2.95×10^-08^ | 0.00005 | 30.739 |
| rs11651755 | C | T | 0.485 | 0.074 | 0.008 | 6.37×10^-22^ | 0.00014 | 92.609 |
| rs11708067 | G | A | 0.239 | -0.097 | 0.009 | 3.22×10^-29^ | 0.00019 | 125.909 |
| rs11925227 | A | G | 0.183 | -0.053 | 0.010 | 1.90×10^-08^ | 0.00005 | 31.596 |
| rs11926707 | T | C | 0.374 | -0.046 | 0.008 | 1.64×10^-08^ | 0.00005 | 31.881 |
| rs12088739 | G | A | 0.090 | -0.088 | 0.013 | 1.05×10^-11^ | 0.00007 | 46.240 |
| rs12299509 | G | A | 0.479 | 0.047 | 0.007 | 1.58×10^-10^ | 0.00006 | 40.925 |
| rs12617659 | T | C | 0.147 | -0.069 | 0.010 | 2.92×10^-11^ | 0.00007 | 44.229 |
| rs12910825 | G | A | 0.360 | 0.052 | 0.007 | 2.82×10^-12^ | 0.00007 | 48.811 |
| rs12945601 | T | C | 0.386 | 0.048 | 0.008 | 1.97×10^-09^ | 0.00005 | 36.000 |
| rs12970134 | A | G | 0.265 | 0.056 | 0.008 | 3.99×10^-12^ | 0.00007 | 48.129 |
| rs13234269 | A | T | 0.493 | -0.058 | 0.008 | 7.76×10^-14^ | 0.00008 | 55.866 |
| rs13239186 | T | C | 0.302 | 0.054 | 0.009 | 2.28×10^-10^ | 0.00006 | 40.210 |
| rs1333039 | G | C | 0.401 | -0.053 | 0.007 | 5.35×10^-13^ | 0.00008 | 52.074 |
| rs13330951 | G | A | 0.488 | -0.046 | 0.008 | 1.81×10^-08^ | 0.00005 | 31.693 |
| rs13389219 | T | C | 0.394 | -0.072 | 0.007 | 1.73×10^-22^ | 0.00014 | 95.194 |
| rs1359790 | A | G | 0.287 | -0.080 | 0.008 | 2.52×10^-23^ | 0.00015 | 99.002 |
| rs1496653 | G | A | 0.205 | -0.077 | 0.009 | 2.36×10^-18^ | 0.00012 | 76.364 |
| rs1552224 | C | A | 0.154 | -0.103 | 0.010 | 1.34×10^-24^ | 0.00016 | 104.809 |
| rs16988333 | G | A | 0.090 | -0.075 | 0.013 | 1.00×10^-08^ | 0.00005 | 32.842 |
| rs17086692 | T | G | 0.313 | -0.047 | 0.008 | 2.71×10^-08^ | 0.00005 | 30.908 |
| rs17168486 | T | C | 0.174 | 0.074 | 0.009 | 2.94×10^-15^ | 0.00009 | 62.309 |
| rs17334919 | T | C | 0.100 | -0.140 | 0.013 | 9.06×10^-28^ | 0.00018 | 119.287 |
| rs17405722 | A | G | 0.074 | 0.087 | 0.015 | 2.54×10^-09^ | 0.00005 | 35.508 |
| rs17411031 | G | C | 0.262 | -0.045 | 0.008 | 2.77×10^-08^ | 0.00005 | 30.864 |
| rs1758632 | C | G | 0.377 | -0.049 | 0.008 | 1.35×10^-09^ | 0.00006 | 36.744 |
| rs17631783 | T | C | 0.263 | -0.049 | 0.009 | 4.45×10^-08^ | 0.00005 | 29.942 |
| rs17791483 | G | A | 0.063 | -0.102 | 0.015 | 3.96×10^-12^ | 0.00007 | 48.146 |
| rs1801214 | C | T | 0.400 | -0.090 | 0.007 | 3.01×10^-34^ | 0.00023 | 148.906 |
| rs1899951 | T | C | 0.123 | -0.112 | 0.011 | 1.10×10^-24^ | 0.00016 | 105.203 |
| rs2191348 | G | T | 0.453 | -0.065 | 0.007 | 4.20×10^-19^ | 0.00012 | 79.772 |
| rs2237892 | T | C | 0.062 | -0.096 | 0.016 | 9.68×10^-10^ | 0.00006 | 37.389 |
| rs2246618 | T | C | 0.307 | 0.051 | 0.008 | 1.01×10^-09^ | 0.00006 | 37.297 |
| rs2261181 | T | C | 0.096 | 0.099 | 0.012 | 6.97×10^-17^ | 0.00011 | 69.680 |
| rs2292662 | T | C | 0.151 | -0.063 | 0.011 | 1.46×10^-08^ | 0.00005 | 32.111 |
| rs2294120 | G | A | 0.456 | -0.044 | 0.008 | 2.05×10^-08^ | 0.00005 | 31.445 |
| rs2296173 | G | A | 0.212 | 0.065 | 0.009 | 7.94×10^-14^ | 0.00008 | 55.820 |
| rs2299383 | T | C | 0.423 | 0.041 | 0.007 | 1.66×10^-08^ | 0.00005 | 31.853 |
| rs243019 | C | T | 0.456 | 0.057 | 0.007 | 1.56×10^-15^ | 0.00010 | 63.550 |
| rs2493394 | G | A | 0.107 | 0.073 | 0.011 | 1.05×10^-10^ | 0.00006 | 41.734 |
| rs2796441 | A | G | 0.416 | -0.072 | 0.007 | 1.19×10^-22^ | 0.00015 | 95.932 |
| rs2820426 | A | G | 0.390 | -0.052 | 0.007 | 9.54×10^-13^ | 0.00008 | 50.936 |
| rs2867125 | T | C | 0.172 | -0.060 | 0.010 | 3.84×10^-10^ | 0.00006 | 39.193 |
| rs2908282 | A | G | 0.177 | 0.055 | 0.009 | 4.30×10^-09^ | 0.00005 | 34.484 |
| rs2925979 | T | C | 0.299 | 0.053 | 0.008 | 7.59×10^-12^ | 0.00007 | 46.870 |
| rs2972144 | A | G | 0.355 | -0.091 | 0.008 | 4.31×10^-34^ | 0.00022 | 148.190 |
| rs340874 | T | C | 0.436 | -0.063 | 0.007 | 9.88×10^-18^ | 0.00011 | 73.536 |
| rs348330 | G | A | 0.367 | 0.049 | 0.008 | 1.83×10^-09^ | 0.00005 | 36.148 |
| rs3756784 | G | T | 0.186 | 0.051 | 0.009 | 2.87×10^-08^ | 0.00005 | 30.796 |
| rs3802177 | A | G | 0.311 | -0.122 | 0.008 | 2.92×10^-52^ | 0.00035 | 231.419 |
| rs459193 | A | G | 0.255 | -0.071 | 0.008 | 1.07×10^-17^ | 0.00011 | 73.381 |
| rs4686471 | T | C | 0.390 | -0.053 | 0.008 | 4.32×10^-11^ | 0.00007 | 43.462 |
| rs4810426 | T | C | 0.097 | 0.073 | 0.013 | 2.34×10^-08^ | 0.00005 | 31.188 |
| rs4823182 | G | A | 0.336 | 0.048 | 0.008 | 3.86×10^-10^ | 0.00006 | 39.184 |
| rs4865796 | G | A | 0.307 | -0.053 | 0.008 | 1.08×10^-11^ | 0.00007 | 46.170 |
| rs516946 | T | C | 0.239 | -0.082 | 0.009 | 3.19×10^-22^ | 0.00014 | 93.976 |
| rs5215 | C | T | 0.360 | 0.068 | 0.007 | 1.58×10^-20^ | 0.00013 | 86.261 |
| rs576674 | G | A | 0.167 | 0.065 | 0.010 | 1.56×10^-11^ | 0.00007 | 45.458 |
| rs6059662 | A | G | 0.337 | -0.045 | 0.008 | 1.65×10^-08^ | 0.00005 | 31.872 |
| rs6066138 | A | G | 0.278 | -0.049 | 0.008 | 2.29×10^-09^ | 0.00005 | 35.708 |
| rs61953351 | T | G | 0.250 | -0.070 | 0.009 | 1.45×10^-14^ | 0.00009 | 59.171 |
| rs622217 | C | T | 0.484 | -0.049 | 0.008 | 3.00×10^-10^ | 0.00006 | 39.674 |
| rs6494307 | G | C | 0.426 | -0.044 | 0.008 | 1.35×10^-08^ | 0.00005 | 32.256 |
| rs6515236 | C | A | 0.249 | -0.050 | 0.009 | 3.05×10^-08^ | 0.00005 | 30.674 |
| rs67232546 | T | C | 0.209 | 0.060 | 0.010 | 5.35×10^-10^ | 0.00006 | 38.543 |
| rs6795735 | T | C | 0.411 | -0.056 | 0.007 | 2.11×10^-14^ | 0.00009 | 58.428 |
| rs687621 | G | A | 0.325 | 0.043 | 0.008 | 1.22×10^-08^ | 0.00005 | 32.460 |
| rs6878122 | G | A | 0.318 | 0.056 | 0.008 | 9.38×10^-13^ | 0.00008 | 50.969 |
| rs7138300 | C | T | 0.443 | 0.044 | 0.007 | 7.61×10^-10^ | 0.00006 | 37.857 |
| rs7144011 | T | G | 0.221 | 0.048 | 0.009 | 1.42×10^-08^ | 0.00005 | 32.155 |
| rs7177055 | G | A | 0.282 | -0.065 | 0.008 | 2.62×10^-16^ | 0.00010 | 67.074 |
| rs7185735 | G | A | 0.397 | 0.106 | 0.007 | 1.99×10^-47^ | 0.00032 | 209.257 |
| rs7240767 | C | T | 0.384 | 0.045 | 0.008 | 2.58×10^-08^ | 0.00005 | 31.001 |
| rs72892910 | T | G | 0.172 | 0.065 | 0.010 | 5.93×10^-11^ | 0.00006 | 42.843 |
| rs735949 | C | T | 0.141 | -0.071 | 0.011 | 1.98×10^-11^ | 0.00007 | 44.991 |
| rs753270 | T | C | 0.416 | -0.053 | 0.008 | 2.33×10^-11^ | 0.00007 | 44.670 |
| rs7561798 | G | A | 0.482 | 0.040 | 0.007 | 2.77×10^-08^ | 0.00005 | 30.864 |
| rs7572970 | A | G | 0.278 | -0.059 | 0.009 | 1.19×10^-11^ | 0.00007 | 45.990 |
| rs7619041 | T | A | 0.487 | 0.043 | 0.008 | 3.28×10^-08^ | 0.00005 | 30.533 |
| rs7651090 | G | A | 0.313 | 0.120 | 0.008 | 1.59×10^-56^ | 0.00038 | 250.972 |
| rs7674212 | T | G | 0.409 | -0.047 | 0.008 | 5.65×10^-10^ | 0.00006 | 38.440 |
| rs7685296 | T | C | 0.279 | -0.051 | 0.008 | 2.81×10^-10^ | 0.00006 | 39.799 |
| rs77258096 | A | C | 0.101 | -0.117 | 0.013 | 2.36×10^-18^ | 0.00012 | 76.366 |
| rs7729395 | T | C | 0.051 | 0.137 | 0.016 | 9.38×10^-18^ | 0.00011 | 73.638 |
| rs7756992 | G | A | 0.267 | 0.130 | 0.008 | 4.35×10^-62^ | 0.00042 | 276.496 |
| rs7786095 | G | A | 0.104 | -0.074 | 0.013 | 8.43×10^-09^ | 0.00005 | 33.174 |
| rs780094 | T | C | 0.387 | -0.069 | 0.007 | 8.65×10^-21^ | 0.00013 | 87.448 |
| rs7845219 | C | T | 0.493 | -0.042 | 0.007 | 4.60×10^-09^ | 0.00005 | 34.353 |
| rs7903146 | T | C | 0.292 | 0.306 | 0.008 | 1.00×10^-300^ | 0.00239 | 1578.251 |
| rs7923866 | T | C | 0.379 | -0.097 | 0.007 | 2.07×10^-39^ | 0.00026 | 172.531 |
| rs7929543 | C | A | 0.083 | 0.083 | 0.014 | 1.97×10^-09^ | 0.00005 | 36.000 |
| rs8068804 | A | G | 0.325 | 0.059 | 0.008 | 5.25×10^-14^ | 0.00009 | 56.635 |
| rs8108269 | G | T | 0.281 | 0.064 | 0.008 | 3.58×10^-16^ | 0.00010 | 66.453 |
| rs825476 | C | T | 0.419 | -0.052 | 0.007 | 7.07×10^-13^ | 0.00008 | 51.525 |
| rs840967 | C | A | 0.394 | 0.050 | 0.008 | 5.21×10^-10^ | 0.00006 | 38.595 |
| rs849135 | G | A | 0.501 | 0.100 | 0.007 | 8.98×10^-44^ | 0.00029 | 192.515 |
| rs853974 | T | C | 0.262 | 0.060 | 0.009 | 8.52×10^-12^ | 0.00007 | 46.643 |
| rs9369425 | G | A | 0.292 | 0.055 | 0.009 | 1.33×10^-10^ | 0.00006 | 41.262 |
| rs963740 | T | A | 0.294 | -0.048 | 0.009 | 2.55×10^-08^ | 0.00005 | 31.022 |
| rs982077 | A | G | 0.434 | 0.045 | 0.007 | 3.14×10^-10^ | 0.00006 | 39.585 |
| rs9844972 | C | G | 0.070 | 0.096 | 0.015 | 1.05×10^-10^ | 0.00006 | 41.724 |
| rs9894220 | G | A | 0.434 | -0.059 | 0.008 | 1.31×10^-13^ | 0.00008 | 54.835 |
| rs993380 | A | G | 0.334 | 0.051 | 0.008 | 3.87×10^-10^ | 0.00006 | 39.178 |
| rs9940149 | A | G | 0.179 | -0.058 | 0.010 | 1.03×10^-09^ | 0.00006 | 37.274 |

Abbreviations: SNP, single nucleotide polymorphisms; EA, effect allele; OA, other allele; EAF, effect allele frequency; Beta, effect size; SE, standard error; R2, proportion of variance in exposure variable explained by SNP; F statistic, indicates the strength of the instrument variables. NA, not applicable.

**Supplemental Table 7.** Priori power calculation for the association estimates in MR analyses

| **Exposure** | **R^2^** | **Outcome** | **Power to detect OR/β of following magnitude** | | | |
| --- | --- | --- | --- | --- | --- | --- |
|  |  |  | OR =1.1  β=0.095 | OR =1.2  β=0.182 | OR =1.3  β=0.262 | OR =1.4  β=0.336 |
| Fasting glucose | 3.20% | Periodontitis | 0.88 | 1.00 | 1.00 | 1.00 |
| HbA1c | 2.30% | Periodontitis | 0.75 | 0.99 | 1.00 | 1.00 |
| T2D | 1.34% | Periodontitis | 0.93 | 1.00 | 1.00 | 1.00 |
| Periodontitis  Periodontitis | 0.038% | HbA1c | 0.31 | 0.80 | 0.98 | 1.00 |
|  | 0.055% | T2D | 0.53 | 0.97 | 1.00 | 1.00 |

Abbreviations: HbA1c, hemoglobinA1c; T2D, type 2 diabetes; R^2^ , proportion of variance in exposure variable explained by SNPs; OR, odds ratio; β, effect size.

**Supplemental Table 8.** Characteristics of the SNPs of BMI.

| **Exposure** | **Confounder** | **SNP** | **EA** | **OA** | **Beta** | ***P*** | **PMID** |
| --- | --- | --- | --- | --- | --- | --- | --- |
| Fasting glucose | BMI | rs10811661 | C | T | 0.021 | 5.00×10^-13^ | 28892062 |
|  |  | rs11603334 | A | G | 0.018 | 4.00×10^-06^ | 25673413 |
|  |  | rs11708067 | A | G | -0.016 | 8.90×10^-09^ | 29273807 |
|  |  | rs11715915 | T | C | -0.022 | 6.11×10^-17^ | UKBB |
|  |  | rs7903146 | C | T | -0.028 | 1.00×10^-18^ | 28892062 |
|  |  | rs9368222 | A | C | -0.041 | 1.15×10^-28^ | 28892062 |
| HbA1c | BMI | rs10774625 | A | G | -0.012 | 4.70×10^-08^ | 29273807 |
|  |  | rs1160334 | A | G | 0.018 | 4.00×10^-06^ | 25673413 |
|  |  | rs11708067 | A | G | -0.016 | 8.90×10^-09^ | 29273807 |
|  |  | rs17747324 | C | T | -0.023 | 7.34×10^-10^ | 25673413 |
|  |  | rs2383208 | A | G | -0.035 | 4.39×10^-18^ | 28892062 |
|  |  | rs9935401 | A | G | 0.070 | 2.08×10^-179^ | UKBB |
| T2D | BMI | rs11257655 | C | T | 0.02142 | 1.69×10^-09^ | 28892062 |
|  |  | rs11708067 | A | G | -0.016 | 8.90×10^-09^ | 29273807 |
|  |  | rs10100265 | A | C | 0.01949 | 2.80×10^-15^ | UKBB |
|  |  | rs12970134 | A | G | 0.043 | 1.20×10^-74^ | 29273807 |
|  |  | rs1063355 | T | G | 0.02223 | 4.00×10^-10^ | 28892062 |
|  |  | rs13330951 | A | G | 0.01782 | 1.37×10^-13^ | UKBB |
|  |  | rs13389219 | C | T | -0.014 | 2.60×10^-10^ | 29273807 |
|  |  | rs1552224 | C | A | 0.021 | 4.50×10^-14^ | 29273807 |
|  |  | rs10811661 | C | T | 0.03554 | 2.93×10^-19^ | 28892062 |
|  |  | rs1899951 | T | C | 0.024 | 6.00×10^-09^ | 28892062 |
|  |  | rs2237892 | C | T | -0.03949 | 3.08×10^-27^ | 28892062 |
|  |  | rs2296173 | A | G | -0.01723 | 4.78×10^-09^ | UKBB |
|  |  | rs2299383 | C | T | -0.01798 | 1.71×10^-13^ | UKBB |
|  |  | rs2867125 | T | C | -0.056 | 1.70×10^-94^ | 29273807 |
|  |  | rs3802177 | G | A | 0.021 | 5.00×10^-10^ | 28892062 |
|  |  | rs5215 | C | T | -0.016 | 3.00×10^-11^ | 28892062 |
|  |  | rs7144011 | G | T | -0.02404 | 9.91×10^-17^ | UKBB |
|  |  | rs7185735 | A | G | -0.07 | 2.82×10^-178^ | UKBB |
|  |  | rs72892910 | G | T | -0.04017 | 4.89×10^-36^ | UKBB |
|  |  | rs7561798 | A | G | -0.014 | 5.74×10^-09^ | UKBB |
|  |  | rs7651090 | A | G | 0.03076 | 8.84×10^-16^ | 28892062 |
|  |  | rs7756992 | A | G | 0.03329 | 5.49×10^-21^ | 28892062 |
|  |  | rs2972144 | G | A | -0.0235 | 1.73×10^-08^ | 25673413 |
|  |  | rs7903146 | C | T | 0.031 | 2.20×10^-42^ | 29273807 |
|  |  | rs7923866 | C | T | -0.02922 | 5.29×10^-11^ | 28892062 |
|  |  | rs8108269 | G | T | -0.02732 | 1.27×10^-13^ | 28892062 |

Abbreviations:HbA1c, hemoglobinA1c; T2D, type 2 diabetes; BMI, body mass index; SNP, single nucleotide polymorphisms; EA, effect allele; OA, other allele; Beta, effect size;

**Supplemental Table 9.** Characteristics of the SNPs used as IVs in the causal association of periodontitis with HbA1c

| **SNP** | **EA** | **OA** | **EAF** | **beta** | **SE** | ***P*** | **R^2^** | **F statistics** |
| --- | --- | --- | --- | --- | --- | --- | --- | --- |
| rs10500684 | A | T | 0.815 | -0.028 | 0.005 | 4.6×10^-07^ | 0.00005 | 25.442 |
| rs11168228 | A | G | 0.691 | 0.021 | 0.005 | 4.5×10^-06^ | 0.00004 | 21.031 |
| rs1130072 | T | C | 0.488 | -0.021 | 0.004 | 6.9×10^-07^ | 0.00005 | 24.631 |
| rs1807019 | T | G | 0.796 | 0.024 | 0.005 | 4.8×10^-06^ | 0.00004 | 20.903 |
| rs4583176 | A | G | 0.244 | 0.023 | 0.005 | 3.7×10^-06^ | 0.00004 | 21.427 |
| rs6117933 | T | C | 0.181 | 0.027 | 0.006 | 1.3×10^-06^ | 0.00005 | 23.464 |
| rs7028167 | A | C | 0.783 | -0.025 | 0.005 | 1.5×10^-06^ | 0.00005 | 23.126 |
| rs922161 | A | C | 0.353 | -0.023 | 0.004 | 1.2×10^-07^ | 0.00006 | 28.100 |

Abbreviations: SNP, single nucleotide polymorphisms; EA, effect allele; OA, other allele; EAF, effect allele frequency; Beta, effect size; SE, standard error; R2, proportion of variance in exposure variable explained by SNP; F statistic, indicates the strength of the instrument variables.

**Supplemental Table 10.** Characteristics of the SNPs used as IVs in the causal association of periodontitis with T2D

| **SNP** | **EA** | | | **OA** | | **EAF** | | **Beta** | | **SE** | ***P*** | | **R^2^** | | **F statistics** | |  |
| --- | --- | --- | --- | --- | --- | --- | --- | --- | --- | --- | --- | --- | --- | --- | --- | --- | --- |
| rs10500684 | | A | T | | 0.815 | | -0.028 | | 0.005 | | | 4.56×10^-07^ | | 0.00005 | | 25.442 | |
| rs11168228 | | A | G | | 0.691 | | 0.021 | | 0.005 | | | 4.52×10^-06^ | | 0.00004 | | 21.031 | |
| rs1130072 | | T | C | | 0.488 | | -0.021 | | 0.004 | | | 6.94×10^-07^ | | 0.00005 | | 24.631 | |
| rs12568187 | | T | C | | 0.565 | | 0.020 | | 0.004 | | | 2.98×10^-06^ | | 0.00004 | | 21.827 | |
| rs1807019 | | T | G | | 0.796 | | 0.024 | | 0.005 | | | 4.83×10^-06^ | | 0.00004 | | 20.903 | |
| rs2978889 | | A | G | | 0.491 | | -0.020 | | 0.004 | | | 1.99×10^-06^ | | 0.00004 | | 22.600 | |
| rs4583176 | | A | G | | 0.244 | | 0.023 | | 0.005 | | | 3.67×10^-06^ | | 0.00004 | | 21.427 | |
| rs62177307 | | T | G | | 0.901 | | 0.035 | | 0.007 | | | 7.54×10^-07^ | | 0.00005 | | 24.473 | |
| rs67216148 | | C | G | | 0.180 | | -0.026 | | 0.006 | | | 2.01×10^-06^ | | 0.00004 | | 22.581 | |
| rs7028167 | | A | C | | 0.783 | | -0.025 | | 0.005 | | | 1.52×10^-06^ | | 0.00005 | | 23.126 | |
| rs77369438 | | T | C | | 0.142 | | 0.028 | | 0.006 | | | 4.67×10^-06^ | | 0.00004 | | 20.967 | |
| rs922161 | | A | C | | 0.353 | | -0.023 | | 0.004 | | | 1.15×10^-07^ | | 0.00006 | | 28.100 | |

Abbreviations: SNP, single nucleotide polymorphisms; EA, effect allele; OA, other allele; EAF, effect allele frequency; Beta, effect size; SE, standard error; R2, proportion of variance in exposure variable explained by SNP; F statistic, indicates the strength of the instrument variables.

**Supplemental Figures**


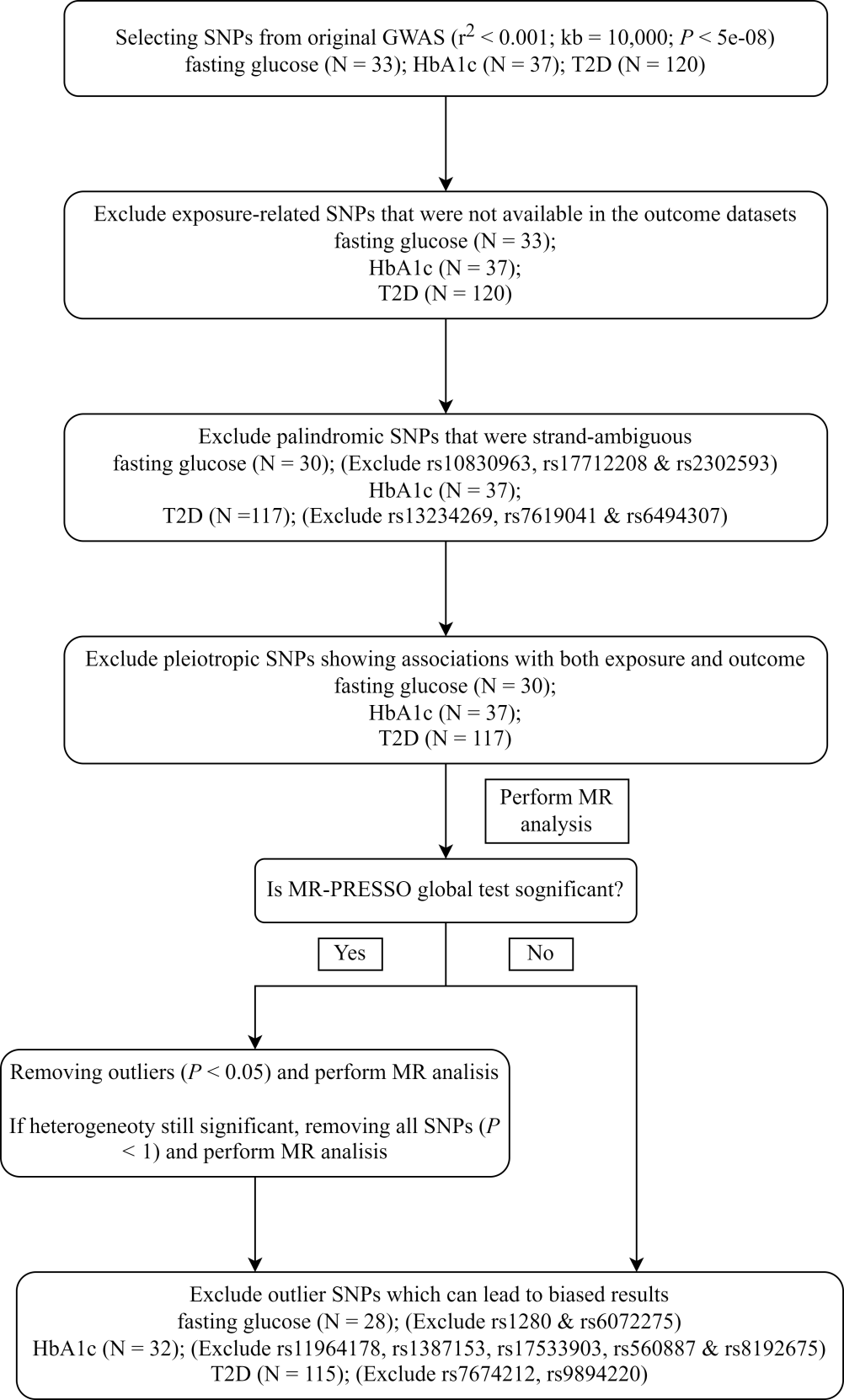


**Supplemental Fig. 1.** Flow chart describing the screening of valid instrumental variable selection of glycemic traits on periodontitis.


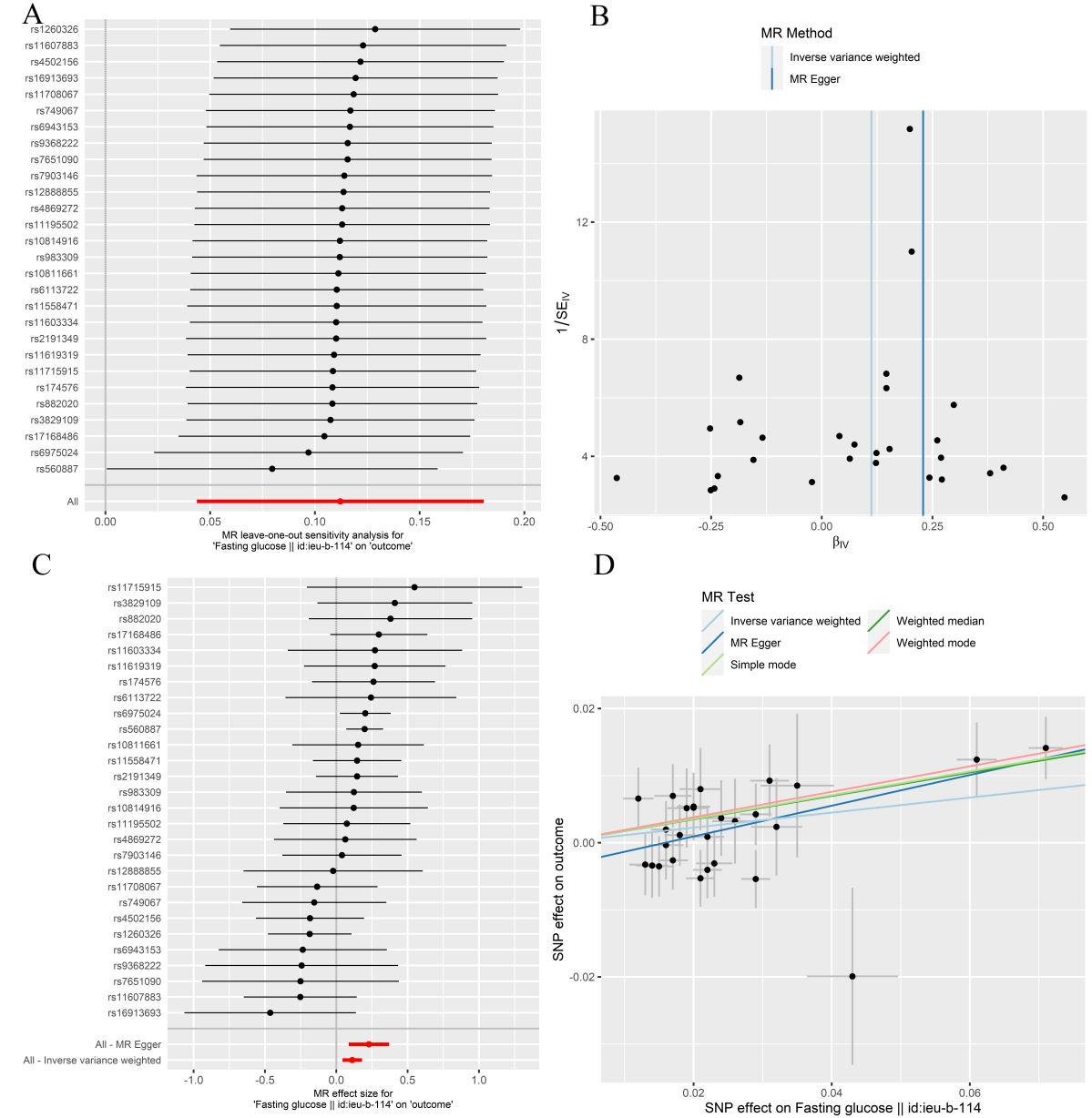


**Supplemental Fig. 2.** Sensitivity analysis of MR estimates on the association of fasting glucose with periodontitis. A, “Leave one out test”; The effect estimates is re-calculated after removal of each SNP to identify if a single SNP is driving the association; B, Funnel plot; the estimates are plotted against the precision of the estimates to test for potential asymmetry. C, Forest plot; It shows the effect estimate of each SNP with 95% confidence interval. D, Scatter plot; SNP-outcome associations are plotted against the SNP-exposure associations to provide the effect estimate for each individual variant. The lines with different colors represent the regression slope fitted by different MR methods.


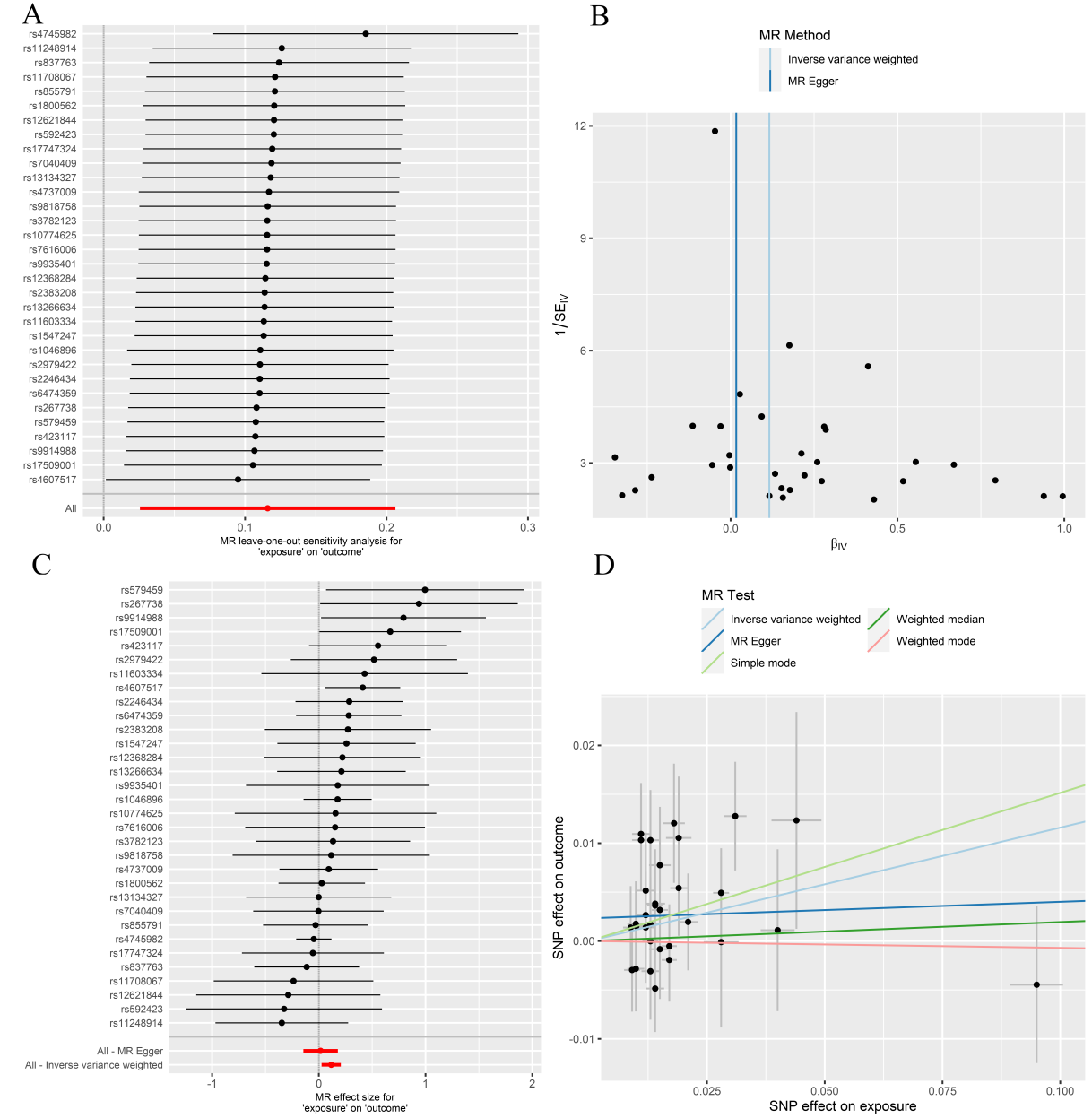


**Supplemental Fig. 3.** Sensitivity analysis of HbA1c on periodontitis. A, “Leave one out test”; The effect estimates is re-calculated after removal of each SNP to identify if a single SNP is driving the association; B, Funnel plot; the estimates are plotted against the precision of the estimates to test for potential asymmetry. C, Forest plot; It shows the effect estimate of each SNP with 95% confidence interval. D, Scatter plot; SNP-outcome associations are plotted against the SNP-exposure associations to provide the effect estimate for each individual variant. The lines with different colors represent the regression slope fitted by different MR methods.


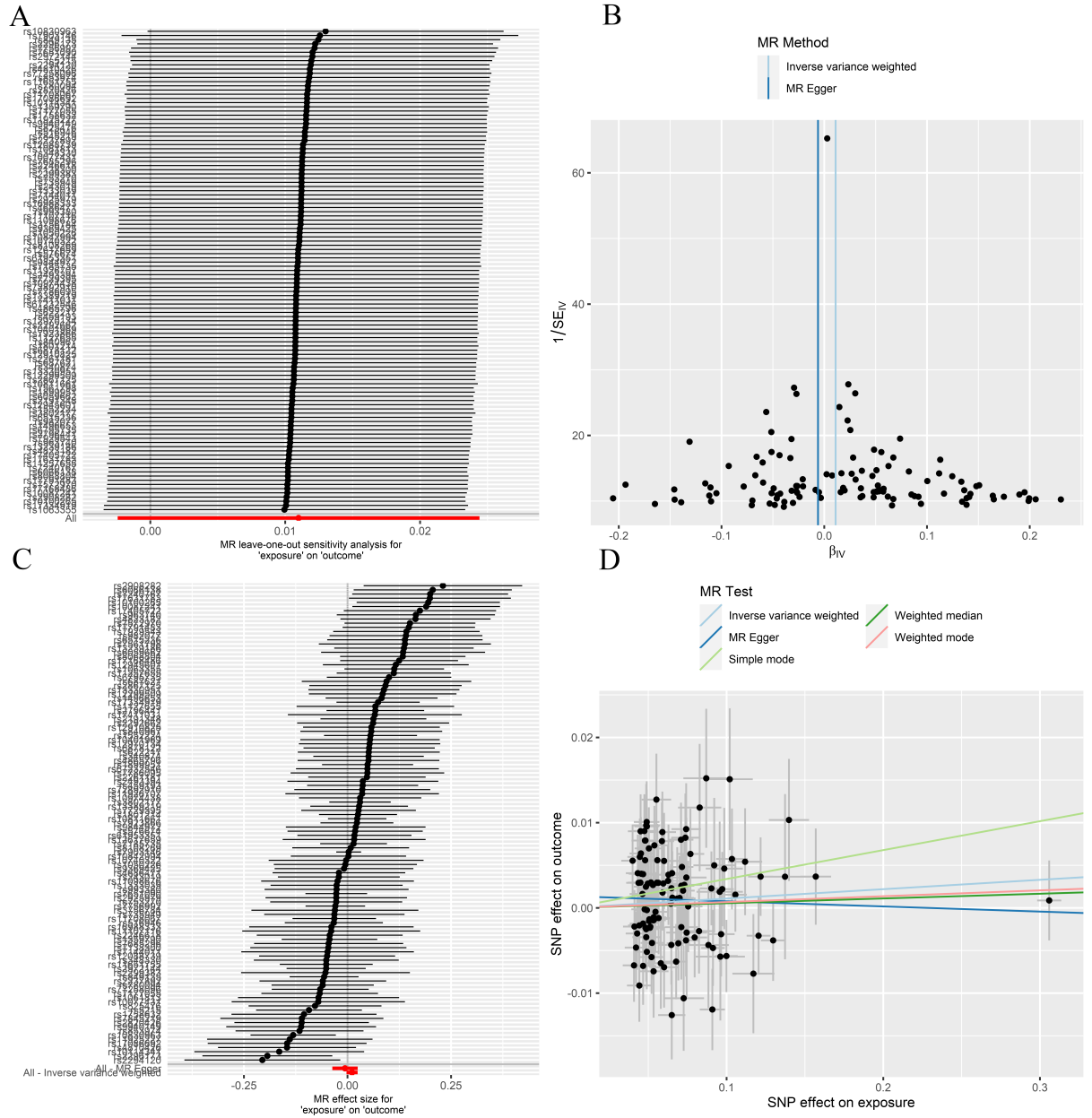


**Supplemental Fig. 4.** Sensitivity analysis of T2D on periodontitis. A, “Leave one out test”; The effect estimates is re-calculated after removal of each SNP to identify if a single SNP is driving the association; B, Funnel plot; the estimates are plotted against the precision of the estimates to test for potential asymmetry. C, Forest plot; It shows the effect estimate of each SNP with 95% confidence interval. D, Scatter plot; SNP-outcome associations are plotted against the SNP-exposure associations to provide the effect estimate for each individual variant. The lines with different colors represent the regression slope fitted by different MR methods.


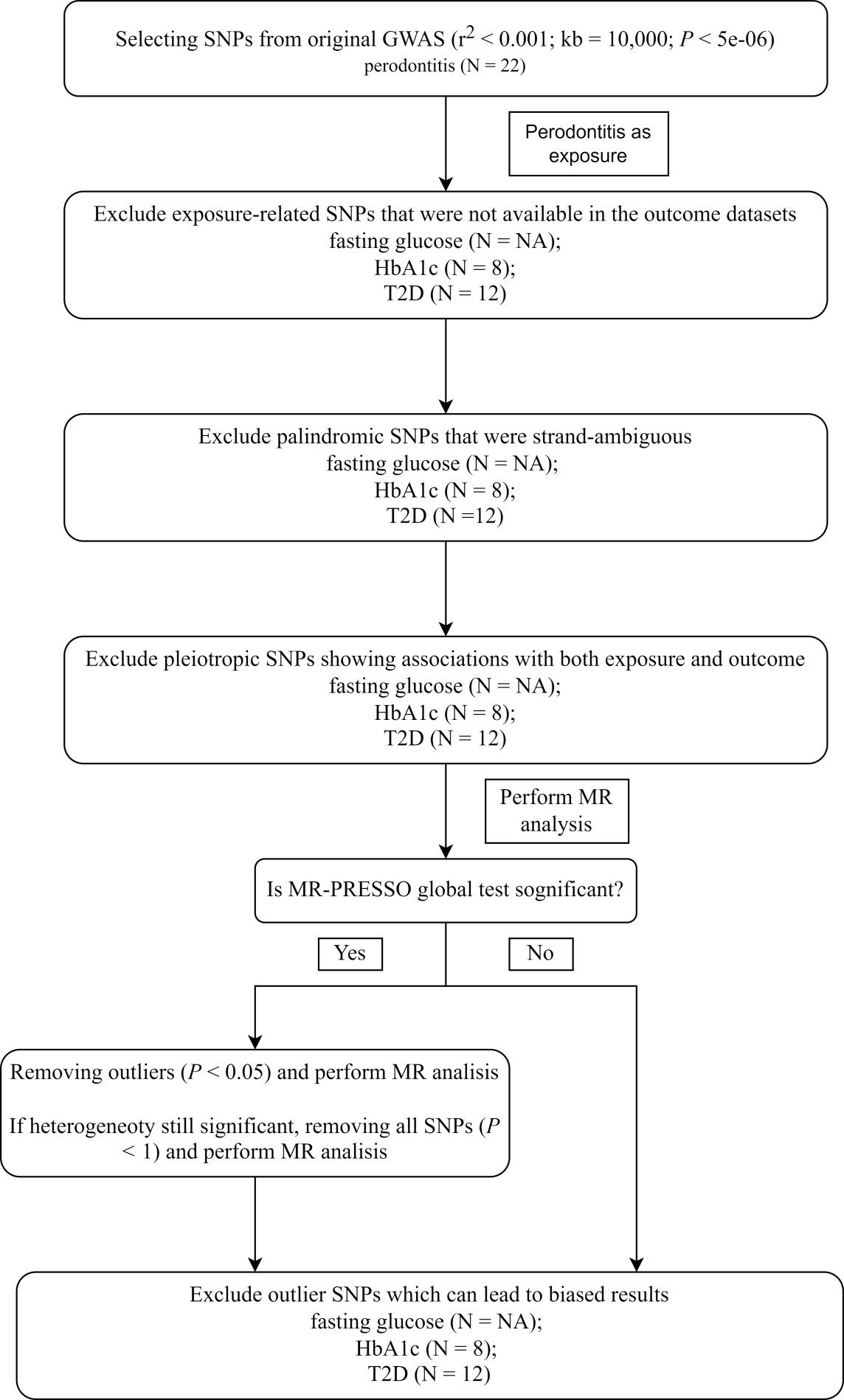


**Supplemental Fig. 5.** Flow chart describing the screening of valid instrumental variable selection of periodontitis on glycemic traits


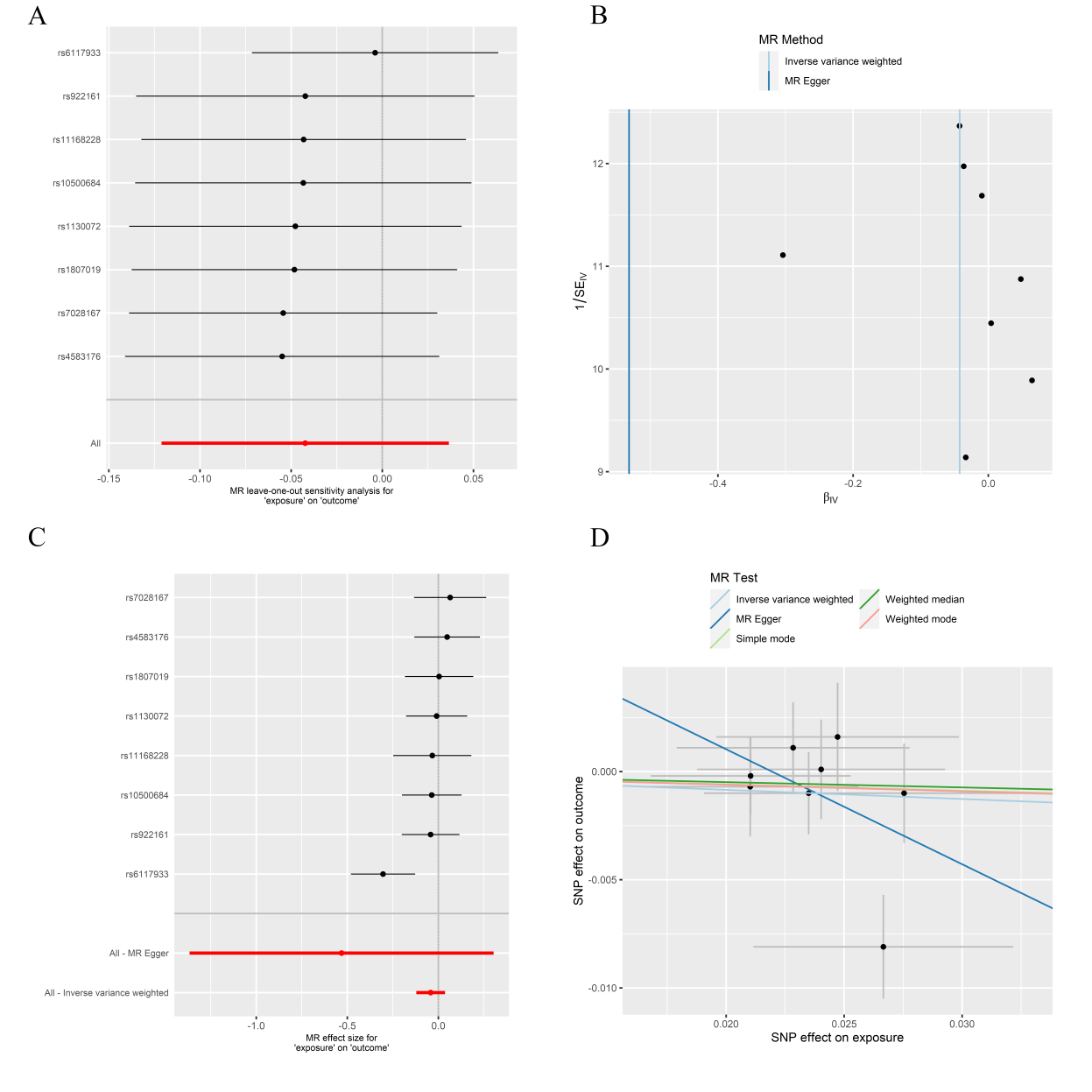


**Supplemental Fig. 6.** Sensitivity analysis of periodontitis on HbA1c. A, “Leave one out test”; The effect estimates is re-calculated after removal of each SNP to identify if a single SNP is driving the association; B, Funnel plot; the estimates are plotted against the precision of the estimates to test for potential asymmetry. C, Forest plot; It shows the effect estimate of each SNP with 95% confidence interval. D, Scatter plot; SNP-outcome associations are plotted against the SNP-exposure associations to provide the effect estimate for each individual variant. The lines with different colors represent the regression slope fitted by different MR methods.


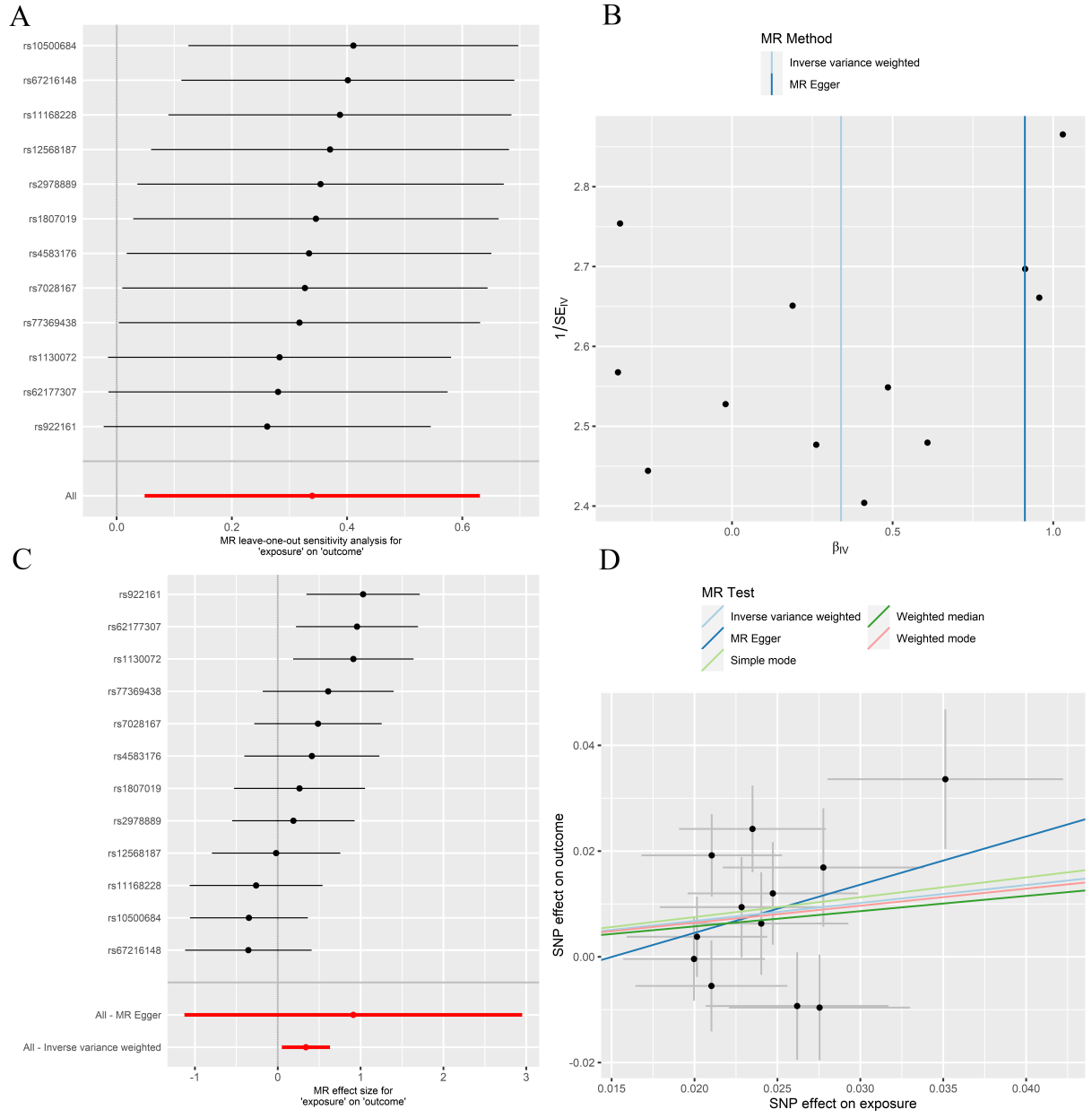
 **Supplemental Fig. 7.** Sensitivity analysis of periodontitis on T2D. A, “Leave one out test”; The effect estimates is re-calculated after removal of each SNP to identify if a single SNP is driving the association; B, Funnel plot; the estimates are plotted against the precision of the estimates to test for potential asymmetry. C, Forest plot; It shows the effect estimate of each SNP with 95% confidence interval. D, Scatter plot; SNP-outcome associations are plotted against the SNP-exposure associations to provide the effect estimate for each individual variant. The lines with different colors represent the regression slope fitted by different MR methods.

**References**

Baumeister SE, Freuer D, Baurecht H, Reckelkamm SL, Ehmke B, Holtfreter B, Nolde M. 2022. Understanding the consequences of educational inequalities on periodontitis: A mendelian randomization study. J Clin Periodontol. 49(3):200-209.

Dupuis J, Langenberg C, Prokopenko I, Saxena R, Soranzo N, Jackson AU, Wheeler E, Glazer NL, Bouatia-Naji N, Gloyn AL et al. 2010. New genetic loci implicated in fasting glucose homeostasis and their impact on type 2 diabetes risk. Nat Genet. 42(2):105-116.

Morris AP, Voight BF, Teslovich TM, Ferreira T, Segre AV, Steinthorsdottir V, Strawbridge RJ, Khan H, Grallert H, Mahajan A et al. 2012. Large-scale association analysis provides insights into the genetic architecture and pathophysiology of type 2 diabetes. Nat Genet. 44(9):981-990.

Prokopenko I, Langenberg C, Florez JC, Saxena R, Soranzo N, Thorleifsson G, Loos RJ, Manning AK, Jackson AU, Aulchenko Y et al. 2009. Variants in mtnr1b influence fasting glucose levels. Nat Genet. 41(1):77-81.

Saxena R, Hivert MF, Langenberg C, Tanaka T, Pankow JS, Vollenweider P, Lyssenko V, Bouatia-Naji N, Dupuis J, Jackson AU et al. 2010. Genetic variation in gipr influences the glucose and insulin responses to an oral glucose challenge. Nat Genet. 42(2):142-148.

Scott RA, Lagou V, Welch RP, Wheeler E, Montasser ME, Luan J, Magi R, Strawbridge RJ, Rehnberg E, Gustafsson S et al. 2012. Large-scale association analyses identify new loci influencing glycemic traits and provide insight into the underlying biological pathways. Nat Genet. 44(9):991-1005.

Shungin D, Cornelis MC, Divaris K, Holtfreter B, Shaffer JR, Yu YH, Barros SP, Beck JD, Biffar R, Boerwinkle EA et al. 2015. Using genetics to test the causal relationship of total adiposity and periodontitis: Mendelian randomization analyses in the gene-lifestyle interactions and dental endpoints (glide) consortium. Int J Epidemiol. 44(2):638-650.

Shungin D, Haworth S, Divaris K, Agler CS, Kamatani Y, Keun Lee M, Grinde K, Hindy G, Alaraudanjoki V, Pesonen P et al. 2019. Genome-wide analysis of dental caries and periodontitis combining clinical and self-reported data. Nat Commun. 10(1):2773.

Wheeler E, Leong A, Liu CT, Hivert MF, Strawbridge RJ, Podmore C, Li M, Yao J, Sim X, Hong J et al. 2017. Impact of common genetic determinants of hemoglobin a1c on type 2 diabetes risk and diagnosis in ancestrally diverse populations: A transethnic genome-wide meta-analysis. PLoS Med. 14(9):e1002383.
